# Engineering sweetness: zeocin-based strain screening to identify high-titer *Komagataella phaffii* clones for brazzein precision fermentation

**DOI:** 10.1007/s10068-026-02150-8

**Published:** 2026-04-10

**Authors:** Jonas Ravn, Vijayendran Raghavendran, Cecilia Geijer

**Affiliations:** 1 Division of Bioeconomy, Department of Food Research and Innovation, RISE Research Institutes of Sweden, Frans Perssons väg 6, 412 76 Gothenburg, Sweden; 2 Department of Life Sciences, Division of Industrial Biotechnology, Chalmers University of Technology, Kemigården 1, 412 96 Gothenburg, Sweden

**Keywords:** Sweet protein, *Pichia pastoris*, Multi-copy gene integration

## Abstract

Brazzein is a heat-stable sweet protein with strong potential as a sugar alternative. Here, we leveraged precision fermentation to produce high titers of recombinant brazzein in the yeast *Komagataella phaffii*. As the brazzein expression cassette included a zeocin resistance marker, we investigated whether high zeocin resistance could serve as a predictive indicator of high brazzein production. A microscale screen across 100–1000 µg mL^−1^ zeocin rapidly identified clones with varying resistance, including the high-resistant Braz_3, which produced 4.5-fold more brazzein than the moderate-resistant Braz_7 in fed-batch bioreactors (61.5 vs. 13.7 mg L^−1^). Long-read genome sequencing revealed that five copies of the expression cassette were integrated into the genome of the high-producing strain compared to a single copy in the moderate-producing strain. Consequently, our results suggest a positive correlation between zeocin resistance and brazzein titers, demonstrating that antibiotic resistance phenotyping can guide strain selection for scalable production of functional food proteins.

**Supplementary Information:**

The online version contains supplementary material available at 10.1007/s10068-026-02150-8.

## Introduction

Microorganisms are increasingly being recognized as viable and sustainable production hosts for food ingredients, proteins, and bioactive compounds, offering solutions to global food security and the climate challenges associated with our current food systems (Graham and Ledesma-Amaro, 2023; Nielsen et al., 2024). The use of engineered microorganisms as cell factories for production of food proteins has expanded through recent developments of new synthetic biology engineering tools and advances in precision fermentation technology (Chai et al., 2022; Chen et al., 2025; Eastham and Leman, 2024). These developments enable the creation of new-to-market designer proteins and molecules with enhanced functionality and health benefits such as better thermostability, gelling capacity, nutritional value and flavor (Rout and Srivastav, 2025). In the future, microorganisms may produce and fulfil many of our dietary needs, including ingredients for sweetening of foods (Malila et al., 2024).

The plant-derived natural sweetener brazzein is a small (54 amino acids, 6.5 kDa), sweet-tasting protein isolated from berry pulp of the West African plant *Pentadiplandra brazzeana* Baillon, also known as *oubli* (from the French word “forgot” referring to the fruit’s intense and attractive sweetness, causing children who eat them forget to go back to their mothers). Brazzein has been found to be 500–2000-fold sweeter than sucrose (on a weight basis), yet nearly calory-free for human consumption (Ming and Hellekant, 1994). Brazzein is highly soluble and remarkably stable due to four disulfide bridges in its protein structure, which help retain its structure and sweetness across a wide pH range (2–10) and at temperatures up to 80–90 °C (Caldwell et al., 1998). These characteristics make it a highly relevant molecule in many food applications that require processing (Ming and Hellekant, 1994; Neiers et al., 2018). Given its history of dietary use and recent confirmation of safety for human consumption (Meetro et al., 2025), brazzein has potential as a healthy sugar alternative to address global diet-related obesity, diabetes, metabolic disorders and address a growing concern for cognitive decline effects from consumption of artificial sweeteners (Gonçalves et al., 2025; Jones et al., 2023).

Because cultivation of brazzein-producing fruits is difficult, and extraction is costly and inefficient (Neiers et al., 2018), biotechnological production using heterologous expression systems in microorganisms is being pursued. Recombinant brazzein production has been successfully demonstrated in the yeasts *Komagataella phaffii* (previously *Pichia pastoris*) (Neiers et al., 2021; Poirier et al., 2012; Rong et al., 2025), *Kluyveromyces lactis* (Lee et al., 2019) and *Saccharomyces cerevisiae* (Kazemi-Nasab and Shahpiri, 2020), as well as in the bacterium *Escherichia coli* (Assadi-Porter et al., 2000). For viable production, the processes must achieve high titers and yields, to ensure economic competitiveness and scalability (Nielsen et al., 2024), and further development is still required before this can be realized for brazzein.

The methylotrophic yeast *K. phaffii* is widely recognized as one of the most significant protein expression systems to date, and the *K. phaffii* strain X-33 is extensively utilized for the production of proteins both in research and in industry (Barone et al., 2023; Cregg et al., 2000). Engineering *K. phaffii* can be achieved through genomic integration of the expression cassette in the alcohol oxidase I (*AOX1*) promoter, which is methanol-inducible and enables tight, controllable regulation of recombinant protein expression. Clonal variation in protein expression levels in this yeast is common, and often caused by differences in gene copy numbers and genomic integration sites of the expression cassettes (Weis, 2019). Therefore, initial screening and selection of strains with high product titers can be highly beneficial for the overall process, especially in production of precision fermented food ingredients that require cost-efficient titers at large scale for viable commercialization (Nielsen et al., 2024). Depending on the integration vector, antibiotic markers such as G418 (geneticin) or zeocin (bleomycin) may accompany the gene of interest upon genomic integration of the linearized vectors by homologous recombination. In these cases, a high antibiotic-resistant strain phenotype may be the result of multicopy integrations and increased heterologous gene expression (Romanos, 1995; Scorer et al., 1994). However, investigations into optimal gene copy numbers in *K. phaffii* indicates that excessively high copy numbers can be detrimental for heterologous expression (Reséndiz-Cardiel et al., 2017). This is because multiple insertions of a foreign gene may impose a high metabolic burden on the host, leading to altered metabolism, impaired secretion, and reduced growth (Zhu et al., 2011). Striking a balance in optimal gene copy engineering is therefore essential.

The aim of this study was to engineer and identify a high-producing *K. phaffii* strain for recombinant brazzein by employing a zeocin-based screening strategy. Transformants carrying the brazzein expression cassette were screened for zeocin resistance across a range of concentrations in microscale cultures, and selected strains were subsequently evaluated for brazzein production both at small scale in shakeflasks and in fed-batch bioreactors to assess the predictive value of the screening approach. To link brazzein expression and zeocin resistance phenotypes to genotypes, long-read whole-genome sequencing was performed on high-, moderate-, and low-zeocin resistant clones to determine gene copy numbers and integration sites of the brazzein and zeocin marker genes.

## Materials and methods

### Yeast strain and plasmid used

The brazzein gene (pBra) from the *P. brazzeana* plant was codon optimized for *K. phaffii* (Supplementary list S1), synthesized by GenScript (Rijswijk, Netherlands), and directly delivered in the pPICAZαA expression vector containing a C-terminal His_6_-tag for heterologous expression in the *K. phaffii* X-33 strain using the EasySelect™ Pichia Expression Kit (Thermo Fisher Scientific, Waltham, MA, USA). The synthesized brazzein cDNA sequence contained a N-terminal glutamine residue (Q1-bra) instead of the rare plant pyroglutamic acid (pyrE), as this substitution has been shown to enhance translation efficiency in yeast while preserving sweetness comparable to the wild-type protein (Poirier et al., 2012).

### Transformation of the *K. phaffii* strain X-33

The pBra_pPICZαA expression vector was linearized with the SacI restriction enzyme, and purified to remove salts and enzymes by addition of 1 × volume of 3M Sodium acetate (pH 5.2) and 2.5 × volume of 96% ice cold ethanol and incubation over night at − 20 °C. Ethanol precipitated DNA was thereafter centrifuged 13,000 rpm for 30 min at 4 °C, washed with 70% ethanol and dried at 37° before dissolving in 50 µL dH_2_O (no resuspension nor vortex). A total of 15 µg of linearized plasmid was used to transform competent *K. phaffii* X-33 cells through electroporation as described by the EasySelect^™^ Pichia Expression Kit manual (Thermo Fisher Scientific, Waltham, MA, USA). Transformants were selected on plates with YPD (1% w/v yeast extract, 2% w/v peptone, 2% d-glucose,) containing 100 µg mL^−1^ Zeocin^™^, 1 M sorbitol and 2% w/v agar agar. Linearized pPICZαA plasmids are designed to integrate into the *AOX1* locus of the genome via homologous recombination. Genomic integration of linearized pBra_pPICZαA vectors into *K. phaffii* X-33 was confirmed by colony PCR with the forward primer: 5′-GCTGAAGCTGTCATCGGTTACTC-3′ and reverse primer: 5′- CAAATGGCATTCTGACATCCTCTTGA-3′ aligning to the α-factor secretion signal and the *AOX1* terminator plasmid parts, respectively (Supplementary Figure S1).

### Strain screening in zeocin containing media

pBra_pPICZαA transformants (only medium-to-large colonies were picked to avoid non-integrants) were inoculated and pre-cultured overnight at room temperature (without shaking) in sterile liquid YPD medium in a 96-well plate, to allow modest yeast growth. 96-well plates containing 250 µL YPD medium with zeocin at 100, 200, 500 and 1000 µg mL^−1^ were then inoculated with 5 µL of pre-culture and growth was monitored for 77 h at 30 °C and 200 rpm using a 96-well plate setup in a Growth-Profiler 960 (EnzyScreen, Heemstede, Netherlands).

### Growth traits data analysis

Growth trait kinetics of transformants in zeocin-containing media were assessed using data generated by the Growth-Profiler 960. Raw output files were processed using a custom R script. Each individual growth curve was smoothed using a mild LOESS regression (span = 0.2) to reduce noise and subsequently adjusted to enforce monotonicity through cumulative maximum transformation. Growth parameters were extracted using the SummarizeGrowth() function from the growthcurver R package (Sprouffske and Wagner, 2016). This function fits a standard logistic growth model and outputs key phenotypic parameters, including Initial population size (n0), Maximum growth rate (r), Generation time (t_gen), Area under the curve (AUC), both logistic fit (auc_l) and empirical (auc_e), Standard deviation (σ) of residuals and an Estimated lag phase, calculated as t_mid − (1/r). The primary phenotype used in downstream analysis was the area under the curve, as it reflects the cumulative biomass production over time. Growth curves with insufficient signal or abnormal parameter estimates were excluded using defined thresholds for flatness and plausibility. Data handling was performed using the tidyverse and dada table in R (version 4.3.2).

### Small scale expression of brazzein

Selected *K. phaffii* X-33 clones were grown in small scale at 20 mL, at 200 rpm in an Innova 44R shaker (orbital diameter 2.5 cm) and 26 °C in 100 mL sterile baffled Erlenmeyer shake flasks covered with sterile mira-cloth to allow aeration in rich buffered glycerol-complex (BMGY) medium (1% w/v yeast extract, 2% w/v peptone, 1.34% w/v yeast nitrogen base with ammonium sulfate without amino acids (YNB), 4 mg mL^−1^ D-biotin, 100 mM potassium phosphate, pH 6.0, 1% v/v glycerol). After 24 h, methanol induction of protein expression was performed by harvesting cells by centrifugation (3000 rpm, 6 min) at room temperature and resuspending cells to an OD_600_ = 1.0 in buffered minimal methanol (BMMY) medium (1.34% w/v YNB, 4 mg mL^−1^ D-biotin, 100 mM potassium phosphate, pH 6.0, 0.5% v/v methanol) for 4 days of induction feeding with 0.5% v/v methanol daily.

### Laboratory scale fed-batch bioreactor expression of brazzein

Selected strains were inoculated into 3 L Labfors 4 bioreactors (Infors HT, Bottmingen, Switzerland) with an initial working volume of 1 L. Bioreactor parameters were set to a temperature of 30 °C, pH 6, stirrer speed at 600–800 rpm and aeration rate of 0.1–0.2 L min^−1^. Bioreactors were inoculated from a 2 mL pre-culture in BMGY medium with 100 µg mL^−1^ zeocin. The strains were first grown in 1 L sterile BMGY medium with 0.79 g L^−1^ complete supplement mixture (MP biomedicals, Santa Ana, CA, USA) and 1.2% (v/v) particulate trace metals (PTM1) for 29 h, with feeding of 10% v/v glycerol at 0.2 mL min^−1^. Cells were starved for 1 h to ensure glycerol depletion before methanol induction. Fed-batch feeding of methanol (0.1–0.2 mL min^−1^) for induction of the *AOX1* promoter driving expression of the brazzein-coding gene was done at a flowrate of 0.1–0.2 L min^−1^ (5–8% of the total flow cycle time) for 20 h, and the final volume reached 1.3 − 1.4 L. Foaming was controlled by adding sterile antifoam B (Sigma-Aldrich, St. Louis, MO, USA) and pH 6.0 was maintained with sterile 1 M potassium hydroxide (KOH). The final culture broth and yeast biomass were harvested through the sample pipe using a Watson-Marlow pump (Watson-Marlow, Rommerskirchen, Germany). OD_600_ measurements were done by bench-top light spectroscopy of 200-fold diluted fermentation broth before initiating methanol induction and after harvesting phases.

### Purification of recombinant brazzein by affinity chromatography

Secreted, recombinant brazzein proteins were purified by immobilized metal affinity chromatography (IMAC) using 2 × 3 mL Ni-Sepharose excel resins (GE Healthcare, Chicago, IL, USA) in drop gravity columns. After loading of the cell-free culture broth, the column was washed with 2 × column volumes of loading buffer (50 mM Tris, pH 8, 250 mM NaCl) before elution of His_6_-tagged proteins with loading buffer containing 250 mM imidazole. Eluted samples (~ 15 mL) were concentrated and buffer exchanged in 25 mM Tris buffer pH 8 with 250 mM NaCl using 3 kDa Amicon^®^ ultra centrifugal spin columns down to 2 mL (Merck, Rahway, NJ, USA) by centrifugation (3000 × *g*, 15 min, repeated 3 ×). Protein purity was evaluated by sodium dodecyl sulfate–polyacrylamide gel electrophoresis (SDS-PAGE). Bradford assay was used to determine protein concentration (Bradford, 1976).

### Proteomic and glycoproteomic characterization of brazzein

Proteomic and glycoproteomic analysis was performed by the Proteomics Core Facility, Sahlgrenska academy at the University of Gothenburg in Sweden. Protein sample (100 µg) in Tris-NaCl pH 8 buffer, 10% glycerol was processed using a modified SP3 digestion method. Sample was reduced in 10 mM dithiothreitol at 56 °C for 30 min and alkylated in 20 mM iodoacetamide at room temperature for 30 min. Washed hydrophobic and hydrophilic Sera-Mag^™^ SpeedBeads (Carboxylate-Modified, Cytiva, Marlborough, MA, USA) were added to the samples with a bead to protein ratio of 10:1. Protein was precipitated on the beads by acetonitrile (final concentration 70%), washed with 80% ethanol and dried at room temperature. Beads were resuspended in 50 mM TEAB and proteins were digested with Trypsin/Lys-C mix [1:40, Promega, Madison, WI, USA] overnight and additional 4 h a second time with new enzyme. The magnetic beads were removed, and peptides collected. Peptides were cleaned with High Protein and Peptide Recovery Detergent Removal Spin Column and Pierce peptide desalting spin columns (both Thermo Fischer Scientific, Waltham, MA, USA) according to the manufacturer's instructions.

The samples were analysed on an Orbitrap Eclipse^™^ Tribrid^™^ mass spectrometer interfaced with an Easy nanoLC 1200 liquid chromatography system (both Thermo Fisher Scientific, Waltham, MA, USA). Peptides were separated on an in-house packed analytical column (35 cm × 75 μm, particle size 3 μm, Reprosil-Pur C18, Dr. Maisch) using a stepped gradient from 5 to 80% acetonitrile in 0.2% formic acid over 30 min at a flow of 300 nL/min. The precursor ion mass spectra were acquired at a resolution of 120 000. As many as possible precursors were isolated with an m/z window of 1.4 and fragmented by higher-energy collisional dissociation (HCD) at 30% during a cycle time of 2 s. Fragment spectra were recorded in the Orbitrap at 30 000 resolution. Raw files were processed and analyzed with Proteome Discoverer (ver 3.0, Thermo Fisher Scientific, Waltham, MA, USA) with Byonic. The data was matched against the Brazzein protein sequence using Byonic as a search engine with a precursor tolerance of 5 ppm and a fragment ion tolerance of 100 ppm. Tryptic peptides were accepted with 2 missed cleavages. Methionine oxidation, Cysteine carbamidomethylation and common N-linked glycan databases were set as a variable modifications up to 8 variants. Raw MS data files were screened for diagnostic oxonium glycan ions, m/z 163.1 and 204.1.

### Strain sequencing, annotation and bioinformatic analysis

Selected strains were whole genome sequenced using Oxford Nanopore Technologies. Strains were first pre-cultured in YPD with 100 µg mL^−1^ zeocin and inoculated in 200 mL sterile YPD in 1000 mL baffled Erlenmeyer flasks at 30 °C for 24 h shaking at 200 rpm. Cells were harvested by centrifugation (3000 rpm, 6 min) and washed twice in 10 mL ice-cold phosphate-buffered saline buffer (pH 7.4) before shipping cells on ice for genomic DNA extraction and long-read Oxford sequencing by Cmbio (Aalborg, Denmark). DNA extraction, sequencing, de novo assembly, gene annotation, gene classification and BLAST analysis can be found in the method section of the sequencing report CMD00707 from Cmbio (Supplementary File 2) together with the complete set of statistical metrics (Supplementary File 3). Please note that the strains in the report are named HZR, MZR and LZR for high-, moderate- and low-zeocin resistant strains, respectively, where the clones numbers match the Braz-(nr) strains. Gene copy numbers of Brazzein and *BleoR* were determined by performing BLAST alignments against the assembled genomes using the respective gene sequences as queries.

## Results and discussion

### Strain construction and screening

Transformation of *K. phaffii* X-33 with the linearized pBra_pPICZαA vector yielded over one hundred zeocin-resistant colonies on selective YPD plates (Supplementary Fig. S1A, B). Colony PCR analysis confirmed successful genomic integration of the brazzein expression cassette in the majority of medium-to-large growing transformants colonies tested (Supplementary Fig. S1B–E).

**Fig. 1:**
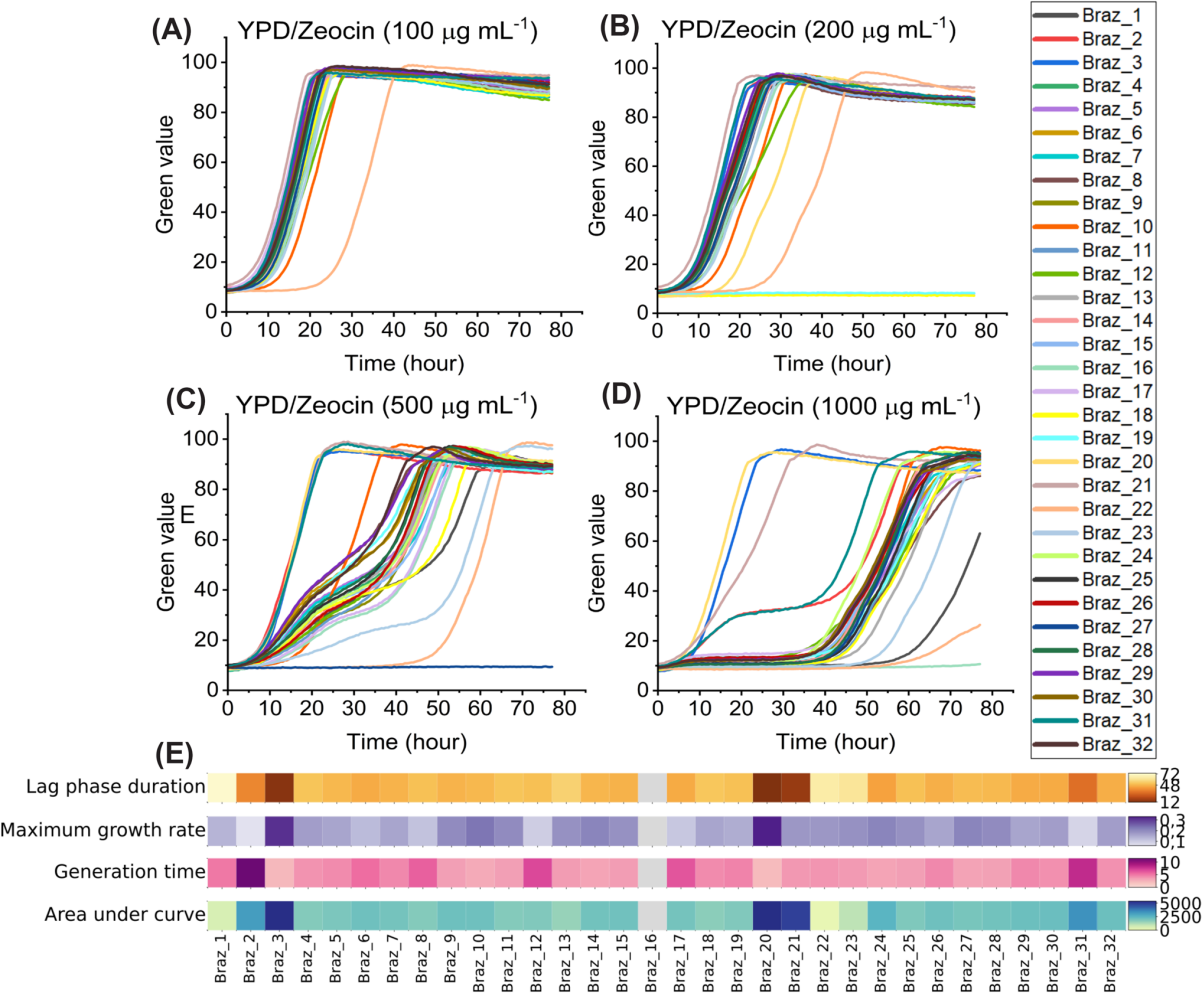
Growth phenotypes of 32 transformants, Braz_1-32, grown in YPD/Zeocin 100 µg mL^−1^ (**A**), YPD/Zeocin 200 µg mL^−1^ (**B**), YPD/Zeocin 500 µg mL^−1^ (**C**) and YPD/Zeocin 1000 µg mL^−1^ (**D**). Green values correspond to growth based on pixel counts determined by a GrowthProfiler 960. Growth profiles are shown as single clones. Heatmaps of the lag phase duration, maximum growth rates, generation time and area under the curve of the 32 clones was calculated from the growth curves of YPD/Zeocin 1000 µg mL^−1^ (**E**)

Screening transformants in micro-scale for high-zeocin resistance may serve as a rapid, phenotypic proxy for optimized gene dosage that can streamline the selection process and bypass the need for laborious qPCR-based copy number quantification during early strain screening for recombinant protein production (Nordén et al., 2011). Therefore, 32 medium-to-large sized colonies were grown in 96 well plates in liquid cultures of YPD and zeocin concentrations ranging between 100 and 1000 µg mL^−1^. Most strains showed similar growth characteristics at 100 µg mL⁻1 zeocin (Fig. 1A). However, with increased zeocin concentrations, growth profiles of transformants became increasingly divergent, with the highest concentration (1000 µg mL⁻1 zeocin) resulting in the strongest variation among strains (Fig. 1B–D). Heatmaps were generated from the area under the growth curves, capturing the combined effects of short lag phases, rapid growth, and high final biomass yields. Heatmaps of other parameters such as lag phase duration, generation time, and maximum growth rate were also generated to compare strain performance in YPD medium containing 1000 µg mL⁻^1^ zeocin (Fig. 1E). Approximately 9% of strains (3 out of 32) showed a high-zeocin resistant growth phenotype, mainly due to their shorter lag phases and faster growth. The high-zeocin resistant strains Braz_3 and Braz_20 displayed lag phases of 13.15 h and 11.56 h, generation times of 2.24 h and 2.08 h and maximum exponential growth rates of 0.31 h^−1^ and 0.33 h^−1^, respectively. The strain Braz_21 (15.95 h, 3.76 h and 0.18 h^−1^) was also highly zeocin resistant but with less efficient growth compared to Braz_3 and Braz_20. Moderate-zeocin resistant strains, including Braz_4, Braz_5 and Braz_7, displayed lag phases of 48–52 h, generation times of 4.0–4.4 h, and maximum growth rates of 0.16–0.17 h^−1^ while low zeocin-resistant strains including Braz_1, Braz_22 or Braz_23 showed markedly extended lag phases of 62–74 h, generation times of 3.7–5.0 h, and maximum growth rates of 0.14–0.19 h^−1^ (Fig. 1E).

### Recombinant brazzein production

High zeocin-resistant phenotypes may be a result of multicopy integration of the expression cassette resulting in increased expression of both the zeocin resistance gene *BleoR* and the brazzein-encoding gene. As an initial screening to investigate if high zeocin-resistant phenotypes may correlate with increased recombinant brazzein titers, protein expression levels of the highest zeocin-resistant strains Braz_3 and Braz_20 were compared to the moderate zeocin-resistant strain Braz_7 in small scale (20 mL, shake flasks) after 96 h of methanol induction. Brazzein production differed between the highly zeocin-resistant strains Braz_3 and Braz_20 and the moderately resistant strain Braz_7, in terms of final concentrations after purification, titers and productivities, where Braz_3 reached the highest titer (0.34 mg L^−1^), similar to Braz_20, and approximately 1.5-fold higher than Braz_7 (Table 1). Based on these initial results, Braz_3 was selected for comparison with Braz_7 in larger-scale 3 L fed-batch bioreactors (1 L working volume), involving 29 h growth on glycerol followed by 20 h methanol induction.

**Table 1:** Overview of recombinant brazzein production from high- and moderate-zeocin resistant strains in shake flasks and bioreactors

Strain	Scale	Zeocin resistance level (1000 µg mL^−1^)	Protein conc. after IMAC (µg mL^−1^)	Titer^1^ (mg L^−1^)	Productivity (mg L h^−1^)
Braz_20	20 mL, shake flask	High	148.1 ± 1.80	0.3	0.16
Braz_3	20 mL, shake flask	High	170.0 ± 3.20	0.34	0.18
Braz_7	20 mL, shake flask	Moderate	118.9 ± 0.15	0.24	0.125
Braz_3	1L, Bioreactor	High	30751.7 ± 683.59	61.5	3.08
Braz_7	1L, Bioreactor	Moderate	6935.2 ± 82.71	13.87	0.69

Protein concentrations were quantified by Bradford assay in triplicates

*IMAC* immobilized metal affinity chromatography

^1^Titers were calculated using final elution volume (2 mL) from His_6_-tag IMAC

**Fig. 2:**
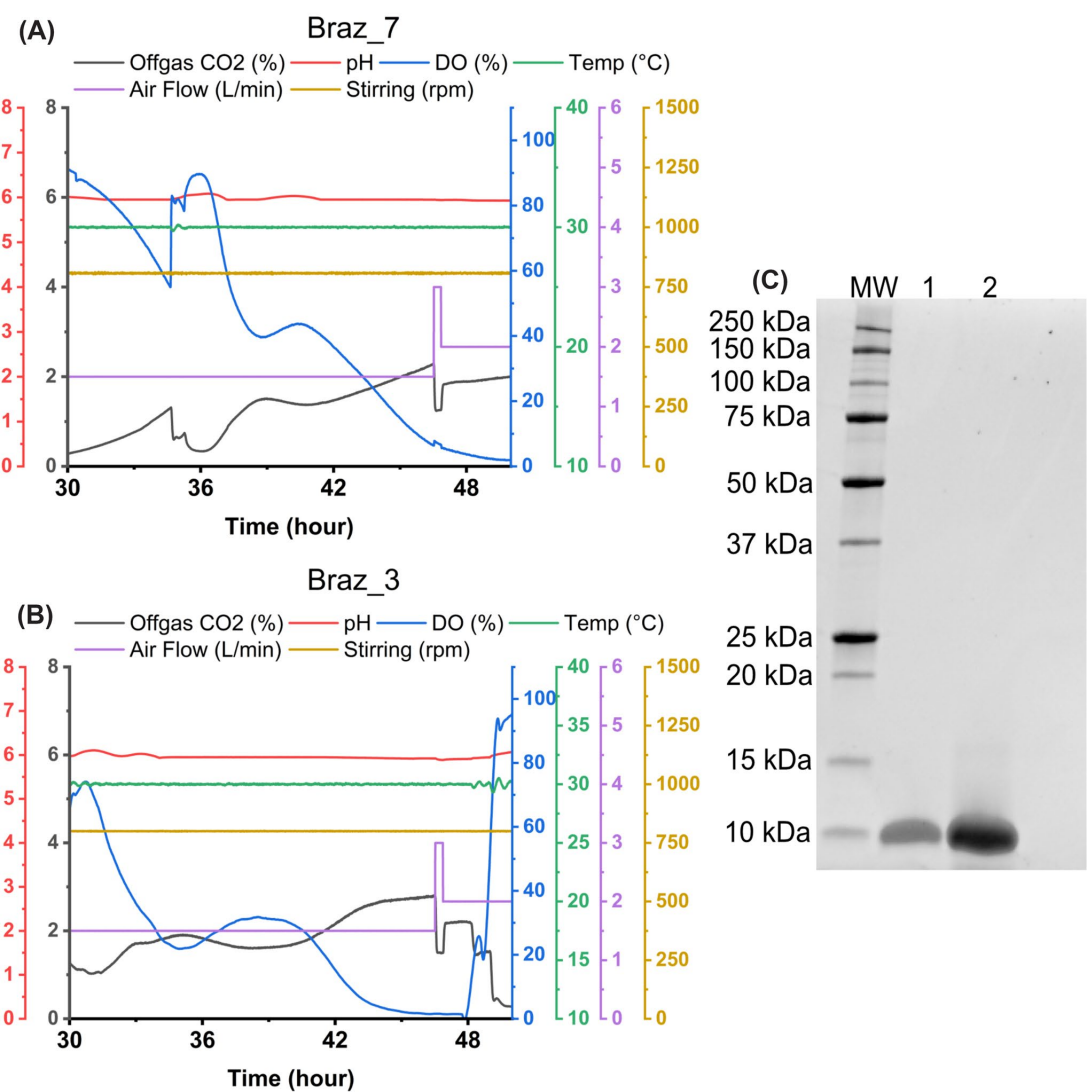
Production of recombinant brazzein in *K. phaffii.* Labfors4 fed batch 3 L bioreactor runs (1 L working volume) of moderate-zeocin resistant strain Braz_7 (**A**) and high-zeocin resistant Braz_3 strain (**B**) indicating off gas CO_2_, pH, dissolved oxygen, temperature, air flow and stirring in buffered minimal methanol medium. SDS-PAGE gel of His_6_-tag purified recombinant brazzein after 100-fold dilution from Braz_7 and Braz_3 strains in 3 L fed batch bioreactors: Lane 1 = Braz_7, lane 2 = Braz_3 (C). *MW* molecular weight; *SDS-PAGE* sodium dodecyl sulfate − polyacrylamide gel electrophoresis

In bioreactors, where high cell densities can be maintained under controlled conditions, the brazzein titer increased further. Braz_3 produced 61.5 mg L⁻^1^ brazzein, 4.5-fold higher than Braz_7 (13.9 mg L⁻^1^), with a corresponding productivity of 3.1 mg L⁻^1^ h⁻^1^ compared to 0.69 mg L⁻^1^ h⁻^1^. Strain differences were also evident in the cultivation profiles: although cell densities were comparable at both the start and end of methanol feeding for Braz_3 and Braz_7 (OD₆₀₀ = 28 and 16 at initiation; 160 and 156 at the end, respectively), their metabolic profiles differed markedly. Braz_3 showed a decline in CO₂ production after ~ 48 h, accompanied by a rise in dissolved oxygen, suggesting that the cells had largely ceased metabolic activity (Fig. 2A, B). In contrast, for Braz_7, the high off-gas CO₂ and low dissolved oxygen at the end of cultivation indicated that methanol feeding could have continued longer as the strain was still metabolically active and likely also still producing brazzein. We can only speculate on the reasons for the respiration arrest observed in Braz_3, although literature provides several possible explanations: High-level heterologous protein synthesis may impose stress on the secretory machinery and trigger the unfolded protein response (Raschmanová et al., 2021). In addition, intensive protein production can transiently deplete metabolic precursors, activating the heat-shock response, slowing growth, and diverting cellular resources toward stress management and maintenance (Glick, 1995). Such precursor depletion may represent a key bottleneck, and supplementation with proteinogenic amino acids could therefore help restore growth in high-producing strains (Heyland et al., 2011). However, exploring such process optimizations was beyond the scope of this study.

Finally, LC–MS/MS analysis of purified brazzein from Braz_3 and Braz_7 confirmed that both strains produced the correct protein sequence. Moreover, glycoproteomics analysis did not detect glycosylation anions from hyper-glycosylation (Supplementary Table S1 and Supplementary Fig. S2, S3), which *K. phaffii* is known to cause during recombinant protein expression (Krainer et al., 2013). These results support the suitability of *K. phaffii* as a production host for functional plant brazzein sweet protein.

### Genome sequencing and gene copy number determination

To investigate the genetic basis underlying differences in zeocin resistance and brazzein production, we performed Oxford Nanopore long-read genome sequencing and genome mapping on eight engineered strains representing high, moderate, and low zeocin-resistance, along with the parental strain. The long-read data enabled precise determination of gene copy numbers and integration loci, allowing us to directly link zeocin resistance phenotypes with the underlying genomic features. Whole genome assembly statistics for all eight strains can be found in Supplementary File 2 and Supplementary File 3, including genome quality, completeness assessments with BUSCO scores and brazzein and *BleoR* gene copy positions.

**Fig. 3:**
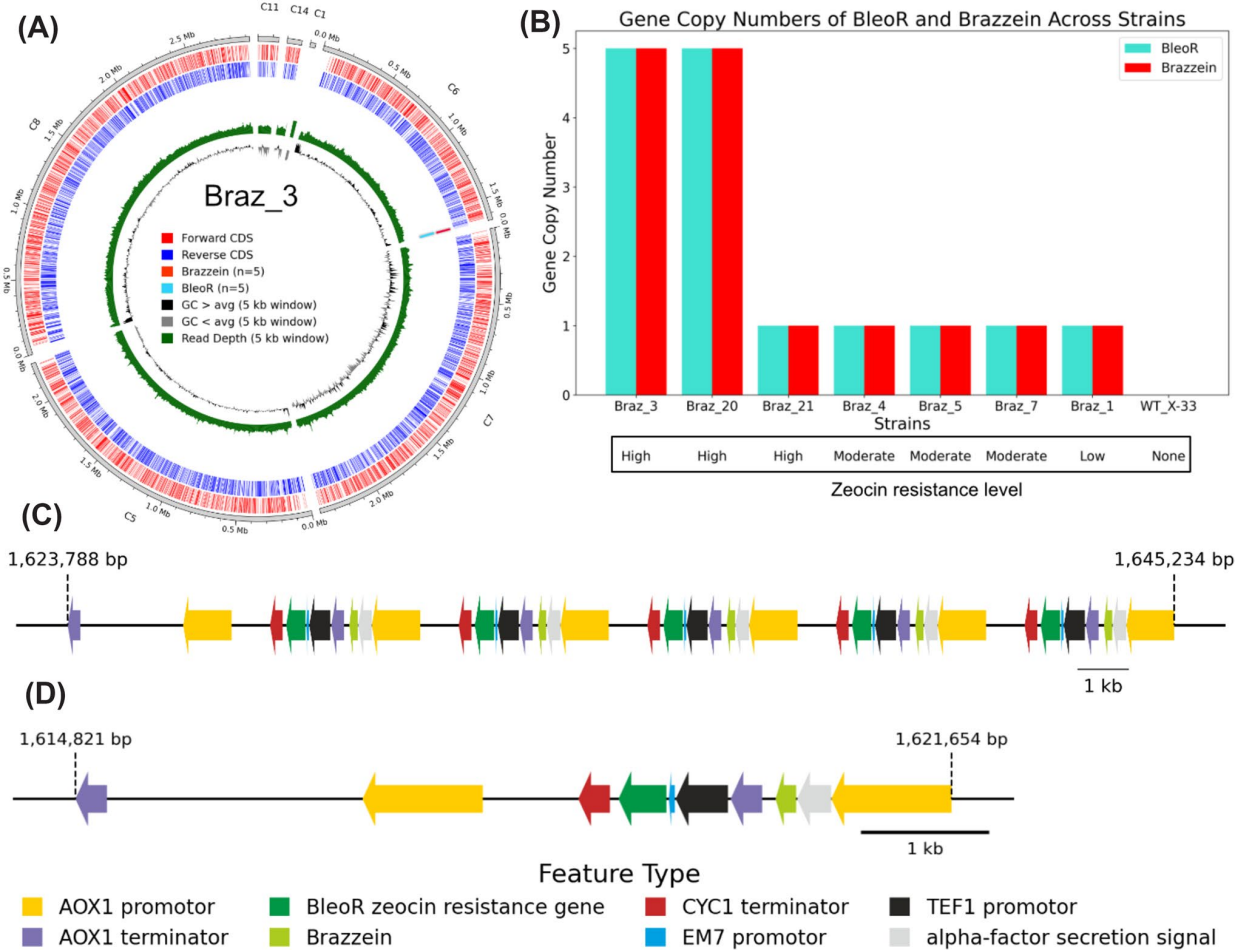
Genetic mapping and gene copy numbers in engineered *K. phaffii* strains. A circular representation of the assembled genome of Braz_3 (**A**). The outermost track shows coding sequences (CDS), followed by tracks marking the genomic locations of BLAST hits for the brazzein and *BleoR* query sequences, providing insight into their distribution across the genome. A depth track illustrates sequencing coverage, capped at 500 × to enhance readability. The innermost track highlights the GC content: regions above and below the average GC content are highlighted to indicate compositional variation. Bar plot with copy numbers of integrated brazzein and *BleoR* genes across different strains (**B**). The sample WT_X-33 is included as a negative control with zero counts for both genes. Specific genomic regions from the circular genome plot, zoomed to show the precise locations of BLAST hits for the brazzein and *BleoR* query sequences for high-zeocin resistant strain Braz_3 (**C**) and moderate-zeocin resistant strain Braz_7 (**D**) including gene features

All engineered strains contained the recombinant gene inserts at the chromosomal region of the *AOX1* promoter loci, except for the low-zeocin resistant strain Braz_1, in which integration occurred within the coding sequence XP_00241935 (Supplementary Fig. S4). This off-target insertion may explain the strain’s poor growth performance. The high zeocin-resistant strains Braz_3 and Braz_20 both had five copies of the expression cassette (including *BleoR* and the brazzein gene) integrated in tandem repeats with identical sequence integration (Fig. 3C). This result aligns with literature, where multicopy transformants with tandem repeat integration have been shown to yield higher recombinant protein yields for example for tetanus toxin fragment C (Clare et al., 1991) and the antimicrobial peptide CC34 (Zhao et al., 2023). In contrast, all moderate zeocin-tolerant strains had one copy insert each (Fig. 3D). Although the number of strains analyzed is too small to draw firm conclusions, these results suggest a positive correlation between zeocin resistance and recombinant brazzein expression. However, the high zeocin-tolerant strain Braz_21 represents an exception to this trend, as it contains only one *BleoR* gene (Fig. 3B). Careful analysis of the expression cassette and genomic insertion site revealed that Braz_21 carries a truncated *5′AOX1* flank lacking 207 bp in a promoter-proximal region, previously shown to strongly influence transcriptional output in *K. phaffii* (Portela et al., 2018). In contrast, the other single copy strains (Braz_4, Braz_5, Braz_7) show only minor deletions of 1–2 bp in the same region (Supplementary Fig. S4). Given that deletions in this region can modulate promoter strength, we speculate that the elevated zeocin resistance of Braz_21 may result from increased *BleoR* expression driven by the truncated *AOX1* flank.

In Braz_3 and Braz_21, the five integrated gene cassettes were placed in tandem. Such tandem arrays have occasionally been reported to undergo “looping-out” through excisional recombination, and the likelihood of such events increases with the length of the tandem array (Le Dall et al., 1994; Lee and Silva, 1997; Zhu et al., 2009). This phenomenon may pose a risk to the genetic stability of *K. phaffii* strains and could lead to decreased production over time. In addition, continuous expression of antibiotic resistance genes imposes an unnecessary metabolic cost. Thus, industrial production strains should ideally be free of such selection markers, which can be achieved using for example CRISPR-Cas9 technology (Gao et al., 2024). Despite these considerations, our results imply that high copy integration of the brazzein gene results in robust secretion of the encoded protein. Moreover, these results are consistent with a recent study in *K. phaffii* that improved brazzein yields through systematic engineering, including promoter and signal peptide optimization and multi-copy integration at neutral genomic sites (Rong et al., 2025).

In conclusion, we developed *K. phaffii* strains for recombinant sweet protein brazzein production focusing on a zeocin-based strain selection. We achieved a 4.5-fold brazzein titer improvement in a high-zeocin resistant strain compared to a moderate-zeocin resistant strain in similar fed-batch bioreactors, likely driven by the multi-copy integration of five tandem brazzein gene repeats at the *AOX1* locus. Although based on a limited number of strains, our results suggest a correlation between zeocin resistance and brazzein titers, indicating that antibiotic resistance phenotyping may serve as a useful proxy for screening production clones and inform strain selection for scalable production of functional food proteins. Moreover, our findings offer valuable guidance for future strain engineering, where marker-free integration of the brazzein gene at multiple, distinct genomic loci may be a promising strategy for stable, high-level production. In the broader context of microbial food production, our study contributes to the growing body of evidence that precision fermentation can deliver scalable, sustainable, and functional food ingredients with minimum environmental and planetary impact.

## Supplementary Information

Below is the link to the electronic supplementary material.

Supplementary file1 (DOCX 787 kb)

Supplementary file2 (PDF 18401 kb)

Supplementary file3 (XLSX 5627 kb)
