# Supplementary material for: Engineering sweetness: zeocin-based strain screening to identify high-titer Komagataella phaffii clones for brazzein precision fermentation
Source: Food Sci Biotechnol. 2026 Apr 10;35(7):1885–94. doi: 10.1007/s10068-026-02150-8 (PMC13172243; doi:10.1007/s10068-026-02150-8)
Supplement: Supplementary file 1 — Supplementary file1 (DOCX 787 kb) [file 10068_2026_2150_MOESM1_ESM.docx]

# Supplementary File 1

**Supplementary list S1**

Codon optimized pBra brazzein DNA sequence

caagataagtgtaagaaagtttatgagaattaccctgtgtcgaaatgccagttagcgaaccaatgcaactacgactgtaaactggataagcatgcaaggtcaggagaatgtttttatgatgaaaagagaaatctacagtgcatctgtgactattgcgagtac

Codon optimized pBra brazzein protein sequence with N-term glutamine (Q1) instead of natural pyroglutamic acid (pyrE).

>ACK76425.1 brazzein, partial [synthetic construct] QDKCKKVYENYPVSKCQLANQCNYDCKLDKHARSGECFYDEKRNLQCICDYCEY


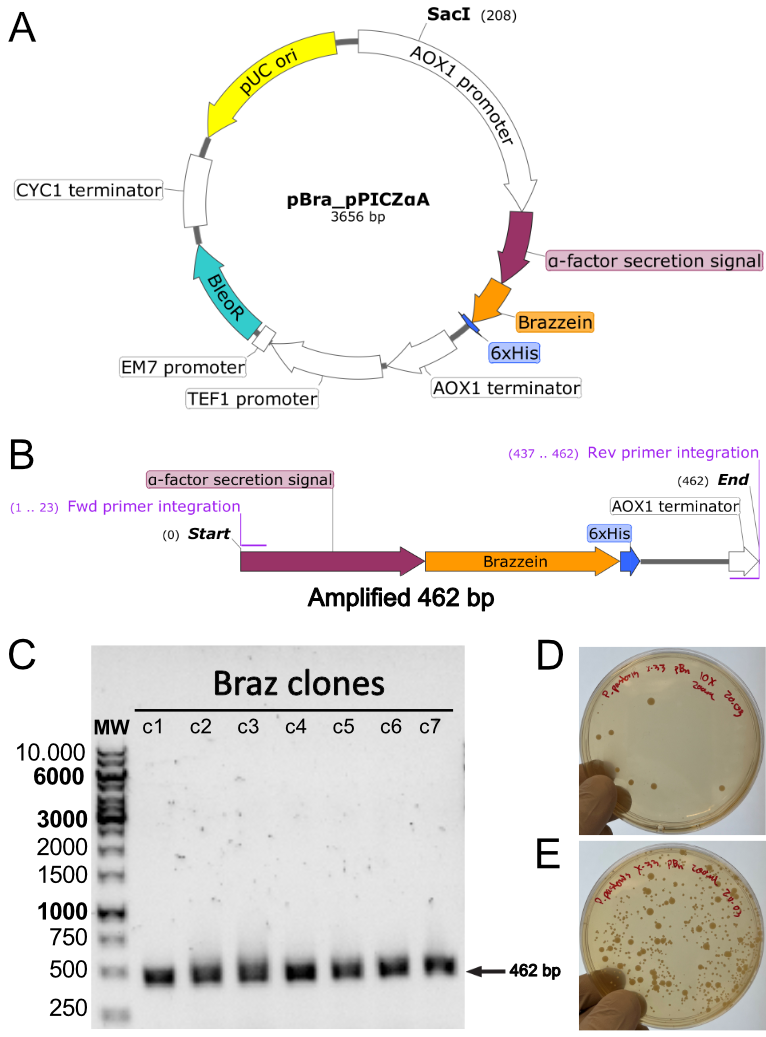


**Supplementary figure S1.** Schematic diagram of the pBra_pPICZαA vector. The sequence coding for brazzein has a C-terminal His_6_-tag for protein purification. The signal peptide α-factor for protein secretion is expressed under the methanol-regulated *AOX1* promoter. *BleoR* encodes a synthetic bleomycin and zeocin resistance factor and placed under control of the *EM7* that drives constitutive expression of zeocin resistance in *E. coli* while the *TEF1* promoter drives expression of the zeocin resistance gene in *K. phaffii*. The *CYC1* transcription termination allows efficient 3´ mRNA processing of the zeocin resistance gene for increased stability. pUC origin allows replication and maintenance of the plasmid in *E. coli.* Zeocin-based strain screening (A)**.** Amplified PCR fragment from colony PCR flanking brazzein gene of interest using forward (Fwd) primer binding to a region in the α-factor secretion signal and reverse (Rev) primer binding to a region in the *AOX1* terminator (B). Agarose gel showing colony PCR amplified fragments from 7 different *K. phaffii* clones (C). Positive colonies on YPD/Zeocin 100 µg mL^−1^ transformants plates from 200 µL 10-fold diluted (D) and non-diluted transformant liquid (E).

**Supplementary Table S1:** Brazzein protein identification by proteomics.

| **Braz_7**  **(81% coverage)** |  |  |  |  |  |  |
| --- | --- | --- | --- | --- | --- | --- |
| **Sequence** | **Modifications** | **# PSMs** | **# Missed Cleavages** | **Theo. MH+ [Da]** | **Confidence: Sequest HT** | **XCorr: Sequest HT** |
| CQLANQCNY  DCK | 3xCarbamidomethyl [C1; C7; C11] | 631 | 0 | 1573,61921 | High | 4,62 |
| NLQCICDYCE  YVDHHHHHH | 3xCarbamidomethyl [C4; C6; C9] | 6 | 0 | 2574,02444 | High | 8,54 |
| RNLQCICDYC  EYVDHHHHHH | 3xCarbamidomethyl [C5; C7; C10] | 1109 | 1 | 2730,12555 | High | 8,21 |
| SGECFYDEK | 1xCarbamidomethyl [C4] | 57 | 0 | 1134,44082 | High | 3,58 |
| SGECFYDEKR | 1xCarbamidomethyl [C4] | 537 | 1 | 1290,54193 | High | 3,79 |
| VYENYPVSK |  | 262 | 0 | 1098,5466 | High | 3,43 |
| VYENYPVSK  CQLANQCNYDCK | 3xCarbamidomethyl [C10; C16; C20] | 622 | 1 | 2653,14797 | High | 4,7 |
|  |  |  |  |  |  |  |
| **Braz_3**  **(82% coverage)** |  |  |  |  |  |  |
| CQLANQCNYDCK | 3xCarbamidomethyl [C1; C7; C11] | 631 | 0 | 1573,61921 | High | 4,77 |
| KVYENYPVSK |  | 6 | 1 | 1226,64156 | High | 4,28 |
| NLQCICDYCE  YVDHHHHHH | 3xCarbamidomethyl [C4; C6; C9] | 1109 | 0 | 2574,02444 | High | 8,45 |
| RNLQCICDYC  EYVDHHHHHH | 3xCarbamidomethyl [C5; C7; C10] | 57 | 1 | 2730,12555 | High | 8,31 |
| SGECFYDEK | 1xCarbamidomethyl [C4] | 537 | 0 | 1134,44082 | High | 3,61 |
| SGECFYDEKR | 1xCarbamidomethyl [C4] | 262 | 1 | 1290,54193 | High | 3,87 |
| VYENYPVSK |  | 622 | 0 | 1098,5466 | High | 3,37 |

A peptide is fragmented multiple times, which allows multiple spectra to be recorded. The number of peptide-spectrum matches (PSM) is called spectra hits. Score Sequest HT: sum of the scores of the individual peptides from the Sequest HT search. It is the sum of all the peptide Xcorr values above the specified score threshold. It is calculated by: 0.8 + peptide_charge X peptide_relevance_factor Where peptide_relevance_factor is a parameter set in the Sequest HT node with a default value of 0.4

**Possible glycosylation sites brazzein**

QDKCKKVYENYPVSKCQLANQCNYDCKLDKHARSGECFYDEKRNLQCICDYCEY

RK cleavage sites, consensus possible N-glycosylation sites

**Results:**

Raw file Eclipse_250624_22

**Supplementary figure S2. Glycoproteomics.** Data base matching against known submitted sequence. PD3.0 was used to search the data using Sequest for ID and Byonic for glycosylation. Sequence coverage of non-glycosylated peptides was 82%. No glycosylation detected using Byonics and N-glycosylation settings for common 20 fungus glycans allowing up to 8 modifications.


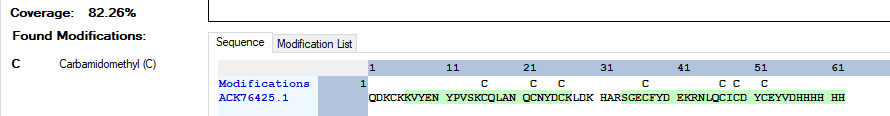


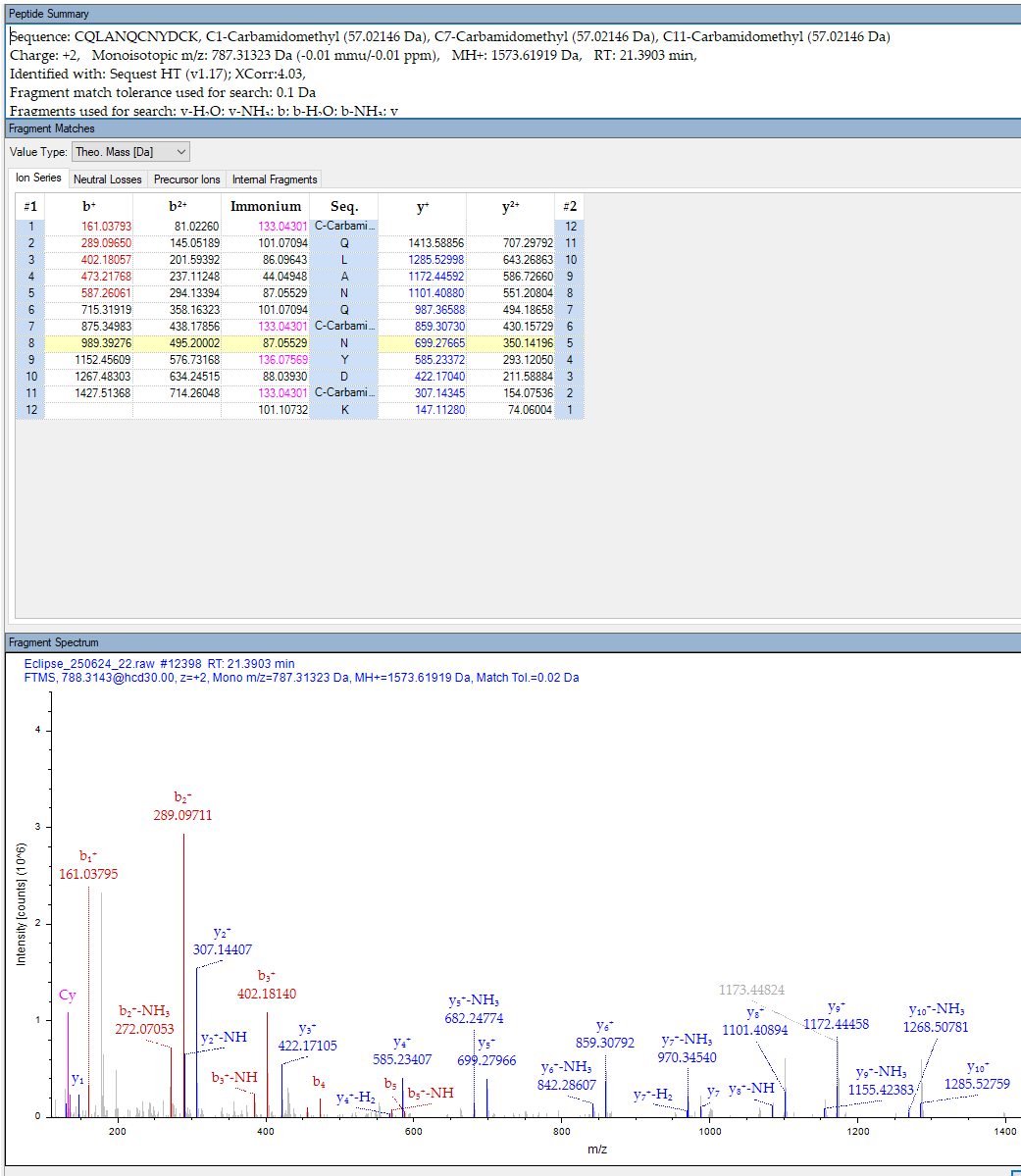


**Supplementary figure S3. Glycoproteomics.** No glycan oxonium ions for HexNAc m/z 204 or 186 in mass spectrum MS2. No glycosylation detected in raw file using RT for fragments ions y10, y6 or y6 from non-modified peptide including the consensus N-glycosylation site CQLANQCNYDCK.

**
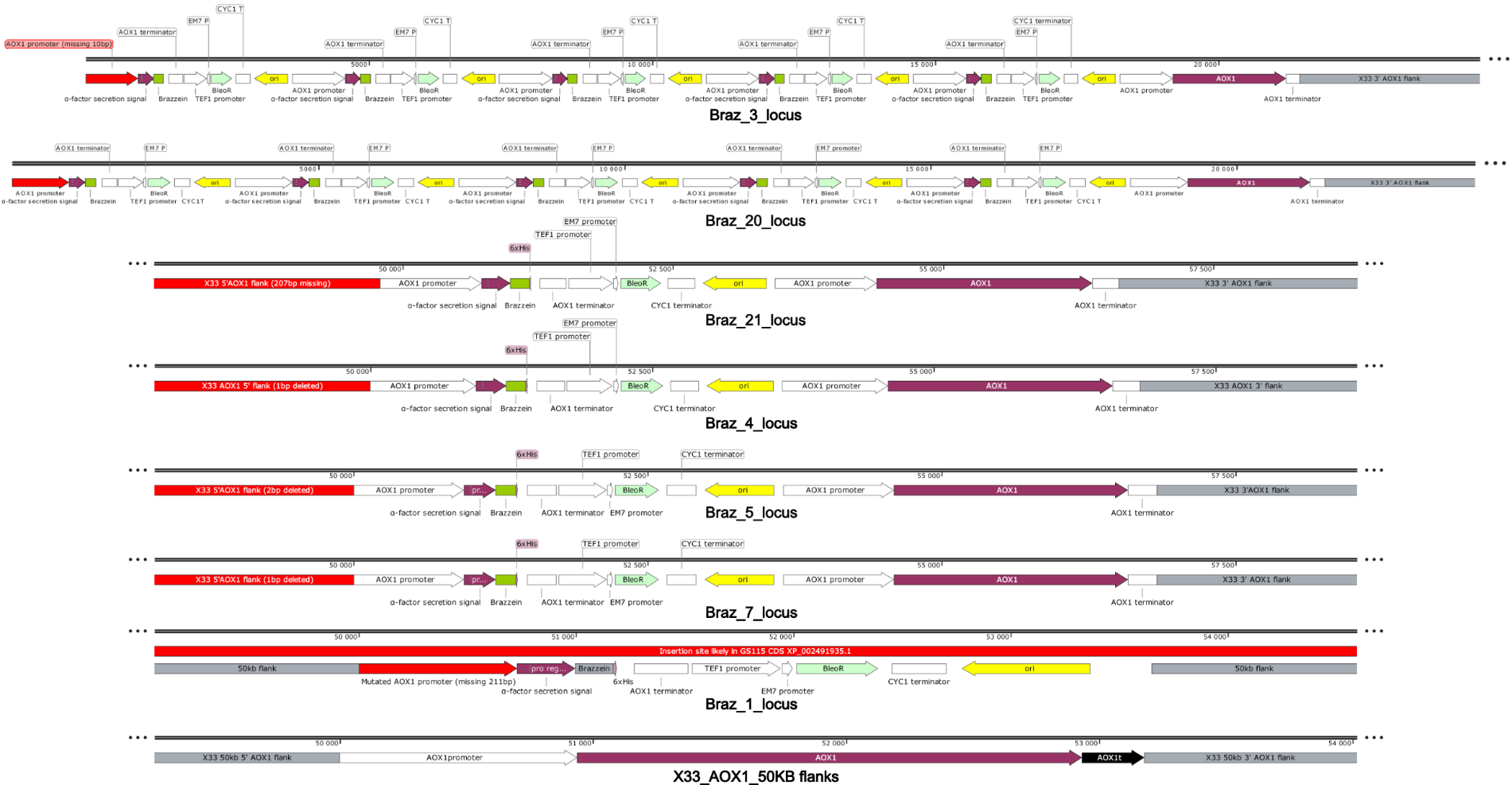
**

**Supplementary figure S4. Integration sites in Braz strains.** Braz_3 and Braz_20 showed similar sequence identity. The five copy integration in *AOX1* locus at the 5’ was at the beginning of the contig, likely therefore missing 10 bp, while the 3’UTR the sequence matches the *AOX1* locus flank, confirming integration in this locus. Single copy integration for Braz_4, Braz_5 and Braz_7 was confirmed in the *AOX1* locus at the 5’*AOX1* flanks had trivial mutations (1-2 bp missing), while the single copy in Braz_21 was truncated in 5’*AOX1* flank with 207bp missing. For the low-zeocin resistant strain Braz_1, single copy was mis-integrated in CDS XP_00241935, while the *AOX1* locus was mutated, missing 211 bp. The non-integrated host strain P-X33 with *AOX1* locus is in the bottom.
