# Supplementary material for: Engineering sweetness: zeocin-based strain screening to identify high-titer Komagataella phaffii clones for brazzein precision fermentation
Source: Food Sci Biotechnol. 2026 Apr 10;35(7):1885–94. doi: 10.1007/s10068-026-02150-8 (PMC13172243; doi:10.1007/s10068-026-02150-8)
Supplement: Supplementary file 2 — Supplementary file2 (PDF 18401 kb) [file 10068_2026_2150_MOESM2_ESM.pdf]

CMD00707 - Yeast genomics

Chalmers University of Technology

September 7th 2025

Version 2025-09-07_16.34.16

Report issued by Rasmus Wollenberg, Ph.D

Contents

**1 Project Scope**	**2**
**2 Data availability**	**3**
**3 Results**	**4**
3.1 DNA Quality Control	4
3.2 Genome Assembly	5
3.3 Genome Quality Assessment	8
3.4 Genome Similarity	10
3.5 Functional Gene Annotation	11
3.6 BleoR and Brazzein Copy Number Evaluation	13
**4 Materials and methods**	**16**
4.1 DNA extraction and quality control	16
4.2 DNA sequencing	16
4.3 <i>De novo</i> assembly	16
4.4 Gene annotation	16
4.5 Classification	16
4.6 BLAST	16
**5 Supplementary information**	**17**
5.1 BLAST Results	17
5.2 Circos Plots	19
5.3 Raw assembly contigs	32
5.4 Quality-filtered contigs	33
5.5 AWS S3 Bucket Access Guide	34
**References**	**35**

1 Project Scope

The objective of this project was to construct 10 high-quality (QV50+) yeast genomes. A total of 10 biomass samples were received from Jonas Ravn at Chalmers University of Technology, and processed using a state-of-the-art workflow designed to maximize data quality and biological insight. This workflow incorporated the latest advancements in Oxford Nanopore sequencing chemistry and followed rigorous scientific standards, as illustrated in Figure 1 [1].

Each sample underwent high molecular weight DNA extraction, followed by long-read library preparation and sequencing. The resulting data were quality-filtered and assembled using a long-read *de novo* assembly pipeline, producing highly contiguous and accurate genome assemblies.

Post-assembly, each genome was analyzed to extract and classify ITS1 and ITS2 regions, using the UNITE database. Functional annotation was performed to identify coding sequences and other genomic features. To assess the presence and genomic context of specific genes of interest, a BLAST analysis targeting *brazzein* and *BleoR* was conducted, enabling evaluation of copy number and genomic location.

This comprehensive approach ensured that each genome was not only of high technical quality but also thoroughly characterized in terms of structure, function, and taxonomic identity.

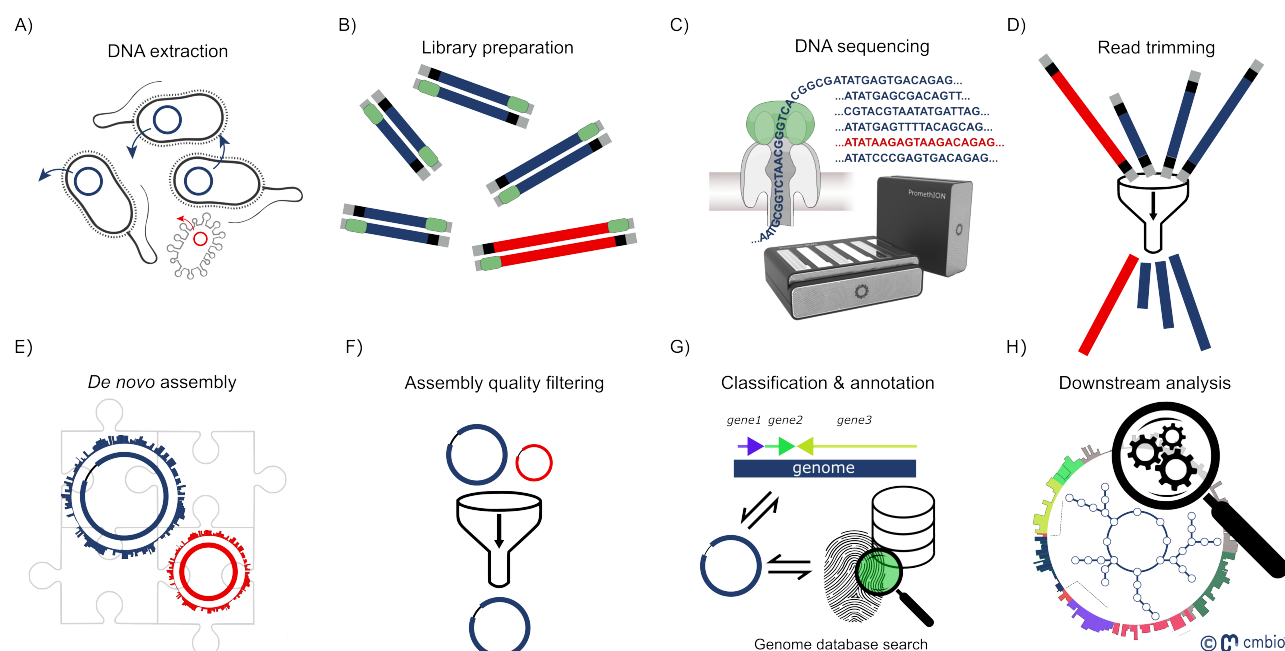

Figure 1: Analysis workflow. Overview of the general genomics workflow. **A)** Total DNA is extracted from the sample biomass. **B)** A DNA sequencing library is prepared and sequenced on a short- or long-read platform. **C)** After quality filtering of the DNA reads, **D)** *de novo* genome assemblies are generated. **E)** Assemblies undergo quality filtering, generating reference-grade genomes. **F)** Each genome is classified and functionally annotated. **G)** High-quality genomes enable downstream bioinformatic analyses, including metabolic pathway reconstruction, functional annotation, and transcriptome profiling.

2 Data availability

All project sequencing and analysis data are hosted in the S3 bucket cmd00707 (region: eu-north-1). The credentials below provide access to the data. For detailed instructions please refer to section 5.5.

- Access Key ID: AKIAWOAVSBU2YLWRE4ZT
- Secret Access Key: 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

The data is organized as follows:

- **/rawData**: Contains unprocessed sequencing data in compressed **.fastq.gz** format, following the DNASense-assigned sequencing ID convention (Table 2).
- **assembly.tar.gz**: Contains all genome assemblies (Tables 3 and 4).
- **annotations.tar.gz**: Contains genome gene annotation files (Table 1).
- **/misc**: A directory holding miscellaneous data tables and report figures.

MD5 checksums are provided for all compressed files to ensure data integrity. Unless otherwise agreed, data will be available for download until September 11th 2025. After this period, data retrieval will incur a fee associated with deep archive access.

Table 1: Functional annotation output description. Overview of the annotation files outputted for the assembled fungal genomes.

Extension	Description
.gff3	A genome annotation file that lists genomic features and their coordinates in a standardized format.
_CDS.fna	Fasta file with coding sequences extracted from the gff3 file.
_proteins.fasta	Fasta file with protein sequences extracted from the gff3 file.
_eggnog.tsv	Tab-delimited tsv file containing functional annotations derived from EggNOG.

3 Results

3.1 DNA Quality Control

Table 2 highlights essential DNA sequencing quality parameters and statistics. DNASense recommends a minimum double-stranded DNA input of 400 ng (50 fmol) for ONT long-read DNA sequencing. DNA should be of high quality and not fragmented to an extent that results in a *subpar* read length distribution. For obtaining high-quality, reference-grade genomes, DNASense recommends a minimum read N50 of 5-7 kbp, a minimum median q-score of 20, and $\geq 50\times$ genome coverage [1,2]. The current ONT sequencing chemistry (V14) supports a modal raw accuracy of Q20 (1 error per 100 bases) or higher.

Table 2: Sequencing statistics. Essential DNA sequencing quality parameters and statistics. **Samplename** and **Sequencing ID** denote customer- and DNASense-assigned sample identification nomenclature, respectively. **Extraction yield (μg)** is the fluorometrically determined quantity of double-stranded DNA. **Data (Mbp)** denotes the sample data yield before (Raw) and after (Filtered) quality filtering. **Read N50 (bp)** is a metric summarizing the read length distribution with half of the data being contained within reads of length N50 and greater. **Quality score** denotes the median Phred-scaled read quality score which is logarithmically related to the error probability.

Samplename	Sequencing ID	Extraction yield (μg)	Raw			Filtered		
			Data (Mbp)	Read N50 (bp)	Quality score	Data (Mbp)	Read N50 (bp)	Quality score
HZR_3	DJ2082-P01-BC01	4.9	1774	11903	23.8	1761	11965	23.9
HZR_20	DJ2082-P01-BC02	21.9	3330	9310	23.7	3301	9367	23.8
HZR_21	DJ2082-P01-BC03	1.2	1794	9077	24.0	1778	9122	24.2
MZR_4	DJ2082-P01-BC04	12.7	3156	8656	23.6	3128	8710	23.8
MZR_5	DJ2082-P01-BC05	2.9	1795	14983	24.1	1785	15041	24.2
MZR_7	DJ2082-P01-BC06	4.8	2150	12785	24.0	2136	12838	24.1
LZR_1	DJ2082-P01-BC07	1.6	1728	8916	24.0	1712	8962	24.1
LZR_22	DJ2082-P01-BC08	0.0	5	1427	23.8	5	1432	24.2
LZR_23	DJ2082-P01-BC09	0.0	5	1503	23.7	5	1503	24.2
WT_P-X33	DJ2082-P01-BC10	2.7	945	14083	23.9	938	14172	24.0

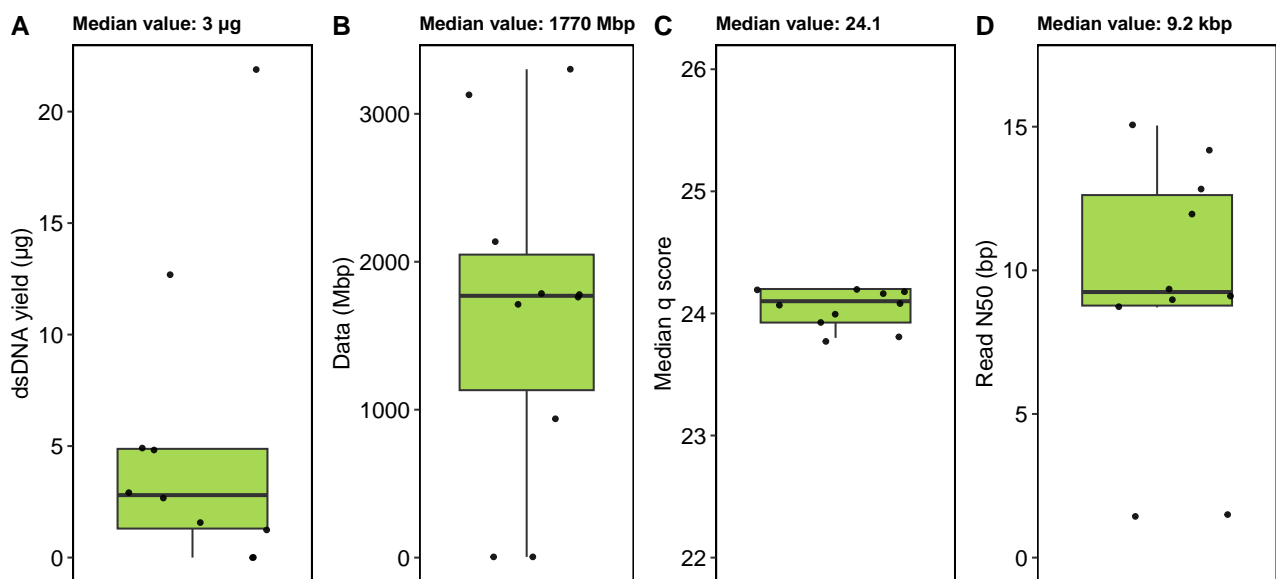

Figure 2: Filtered sequencing data statistics. **A)** Fluorometrically determined double-stranded DNA extraction yield, **B)** Data (Mbp) above a Q20 threshold, **C)** Modal Phred-scaled quality score and **D)** median read N50 (bp).

3.2 Genome Assembly

Table 3 summarizes the results of the whole-genome *de novo* assembly workflow. This process provides the foundation for downstream analyses by reconstructing genomes from sequencing data. Ideally, the workflow produces complete, telomere-to-telomere chromosomes along with any co-occurring extrachromosomal elements, such as circular plasmids and viral DNA. However, repetitive genomic regions can impact assembly contiguity, complicating genome reconstruction.

If the input sample is contaminated or sequenced at extremely high coverage, the assembled and contiguous elements (contigs) may reflect multiple co-assembled genomes or residual contigs from background DNA or contaminants (e.g., kit-derived DNA). Contaminant genomes or contigs can often be filtered out before further analysis, provided the assembly meets quality and contiguity thresholds. Table 4 presents key assembly statistics after filtering out short and low-quality contigs.

Table 3: Raw assembly metrics. This table summarizes genome assembly statistics prior to any quality or contamination filtering. **Samplename** refers to the customer-assigned sample identifier. **Assembly size (Mbp)** indicates the total length of the raw assembly in megabase pairs. **Total contigs**, **Circular contigs**, and **Linear contigs** represent the counts of all contigs and their respective topologies. **Circular fraction** and **Linear fraction** indicate the proportion of the total assembly length composed of circular and linear contigs. **Contig N50 (Mbp)** and **Contig N90 (Mbp)** are metrics describing the contig length distribution, where N50 and N90 represent the minimum contig length needed to cover 50% and 90% of the total assembly, respectively. **GC (%)** denotes the average guanine-cytosine content across the assembly. **Depth** refers to the average sequencing coverage per base in the assembled genome.

Samplename	Assembly size (Mbp)	Total contigs	Circular contigs	Linear contigs	Circular fraction	Linear fraction	Contig N50 (Mbp)	Contig N90 (Mbp)	GC (%)	Depth
HZR_20	9.5	9	1	8	0	1	2.4	1.6	41	346
HZR_21	9.5	6	1	5	0	1	2.4	1.7	41	186
HZR_3	9.5	7	1	6	0	1	2.4	1.6	41	184
LZR_1	9.5	7	2	5	0	1	2.4	1.7	41	179
MZR_4	9.5	13	2	11	0	1	2.4	1.7	41	329
MZR_5	9.5	6	1	5	0	1	2.4	1.7	41	187
MZR_7	9.5	9	3	6	0	1	2.4	1.7	41	223
WT_P-X33	9.5	8	0	8	0	1	2.4	1.8	41	86

Table 4: Quality-filtered assembly statistics. **Samplename** denotes customer-assigned sample identification nomenclature. **Samplename** denotes the customer-assigned sample identifier. **Genome size (Mbp)** and **Contigs** represent the total size of the filtered assembly in megabase pairs and the number of contiguous DNA elements, respectively. **Circular contigs** and **Linear contigs** indicate the number of contigs classified by topology, while **Circular fraction** and **Linear fraction** show their relative proportions. **Contig N50 (Mbp)** and **Contig N90 (Mbp)** are metrics describing the contig length distribution, where N50 and N90 represent the minimum contig length needed to cover 50% and 90% of the total assembly, respectively. **GC (%)** denotes the average guanine-cytosine content across the assembly. **Depth** refers to the average sequencing coverage per base in the assembled genome. **UNITE ITS2** indicates the taxonomic classification results based on extracted internal transcribed spacer (ITS) region 2, using the UNITE database.

Samplename	Genome size (Mbp)	Contigs	Circular contigs	Linear contigs	Circular fraction	Linear fraction	Contig N50 (Mbp)	Contig N90 (Mbp)	GC (%)	Depth	UNITE ITS2
HZR_20	9.5	7	1	6	0	1	2.4	1.6	41	346	Komagataella_kurtzmanii,Komagataella_phaffii
HZR_21	9.5	6	1	5	0	1	2.4	1.7	41	186	Komagataella_kurtzmanii,Komagataella_phaffii
HZR_3	9.5	7	1	6	0	1	2.4	1.6	41	184	Komagataella_kurtzmanii,Komagataella_phaffii
LZR_1	9.5	6	1	5	0	1	2.4	1.7	41	180	Komagataella_kurtzmanii,Komagataella_phaffii
MZR_4	9.5	6	1	5	0	1	2.4	1.7	41	329	Komagataella_kurtzmanii,Komagataella_phaffii
MZR_5	9.5	6	1	5	0	1	2.4	1.7	41	187	Komagataella_kurtzmanii,Komagataella_phaffii
MZR_7	9.5	6	1	5	0	1	2.4	1.7	41	223	Komagataella_kurtzmanii,Komagataella_phaffii
WT_P-X33	9.5	5	0	5	0	1	2.4	1.8	41	86	Komagataella_kurtzmanii,Komagataella_phaffii

3.3 Genome Quality Assessment

The quality of an assembled genome influences downstream analyses and the conclusions drawn from them. Many factors affect genome assembly quality, including sample quality (e.g., biomass age and degree of contamination), the quality of the DNA extract, sequencing technology (longer reads result in less ambiguity), the complexity of the sequenced genome (ploidy and repeat length), and the amount of sequencing data. A common approach to assessing genome assembly quality is to estimate the completeness and redundancy of genomic data using universal marker genes or single-copy orthologs. The number of universal single-copy orthologs is lineage-dependent, so comparisons should be made within the closest available lineage in the database.

BUSCO (Benchmarking Universal Single-Copy Orthologs) is widely used for this purpose, classifying ortholog matches as complete (full-length), fragmented (partial), or missing. Each match is further distinguished as either single-copy or duplicated. While duplicated BUSCOs can reflect genuine gene duplications or polyploidy, excessive duplication often points to assembly artifacts or contamination. BUSCO scores are expressed as percentages, where higher completeness and lower duplication typically indicate better genome quality.

Figure 3 highlight key assembly metrics for the quality-filtered fungal genomes. The complete set of metrics is available in the supplementary file CMD00707_genome_stats.xlsx. Of the 8 assembled genomes, 0 exceeded the 5 % BUSCO multicopy (contamination) threshold.

Although DNASense recommends a BUSCO completeness score of at least 95 %, interpretation should consider the novelty of the organism and the degree of representation in the BUSCO reference. For more comprehensive guidance on interpreting BUSCO results, refer to the original publications [3,4] or consult the official [BUSCO manual](#).

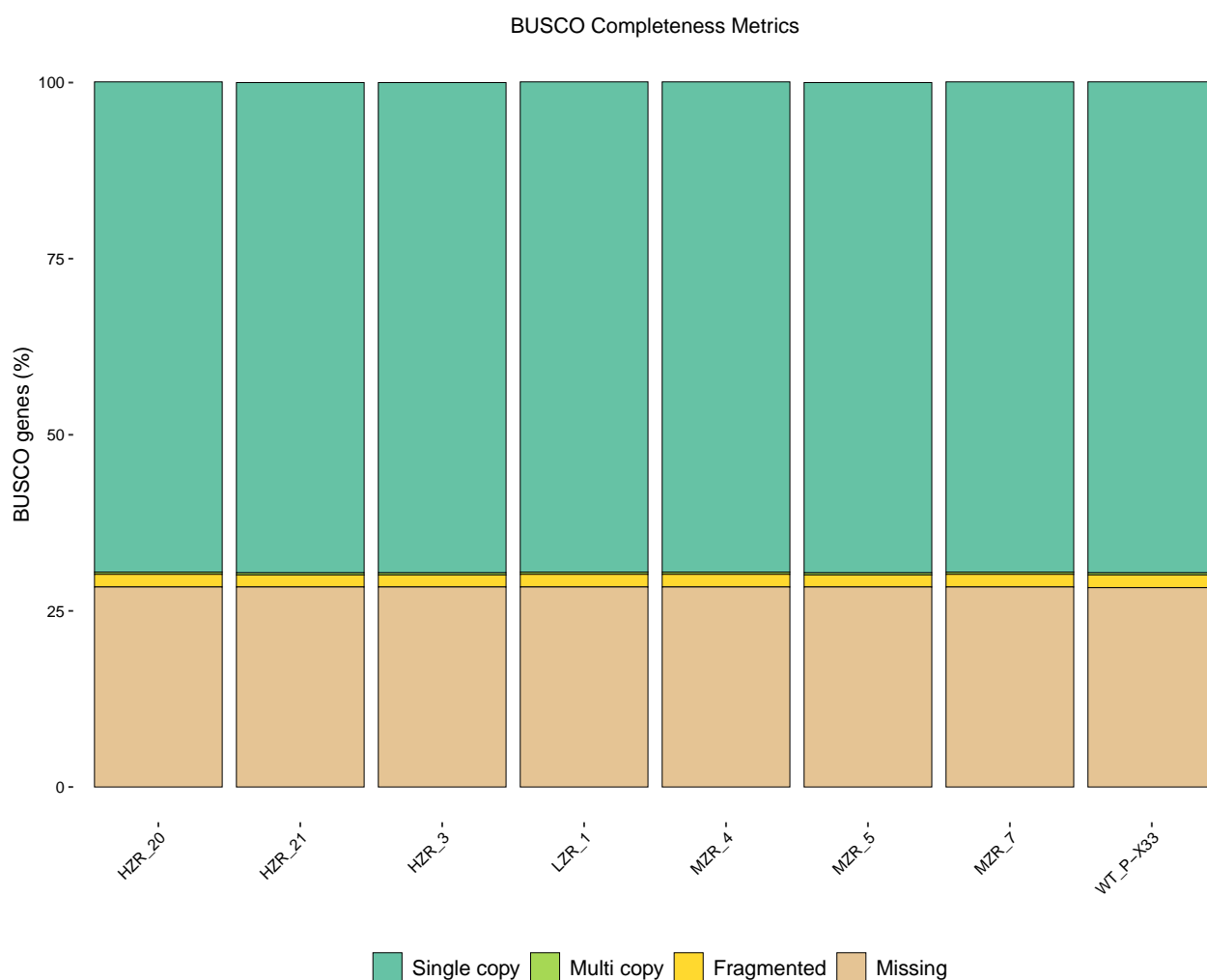

Figure 3: Genome quality assessment. BUSCO plot depicting the proportions of BUSCOs classified as complete and single-copy, complete and duplicated, fragmented, or missing. The y-axis represents the proportion of BUSCOs (in percentage), while the x-axis lists the genome assemblies.

3.4 Genome Similarity

Average nucleotide identity (ANI) is a well-recognized measure of relatedness. Although no universally accepted species-level threshold exists for fungi, ANI serves as a supporting metric in fungal taxonomy and for delineating relatedness between sequenced isolates [5]. Figure 4 shows pairwise ANI values for the assembled genomes, highlighting the degree of relatedness. ANI values below approximately 80% are not shown, as the algorithm assumes a probabilistic identity cutoff of 80%.

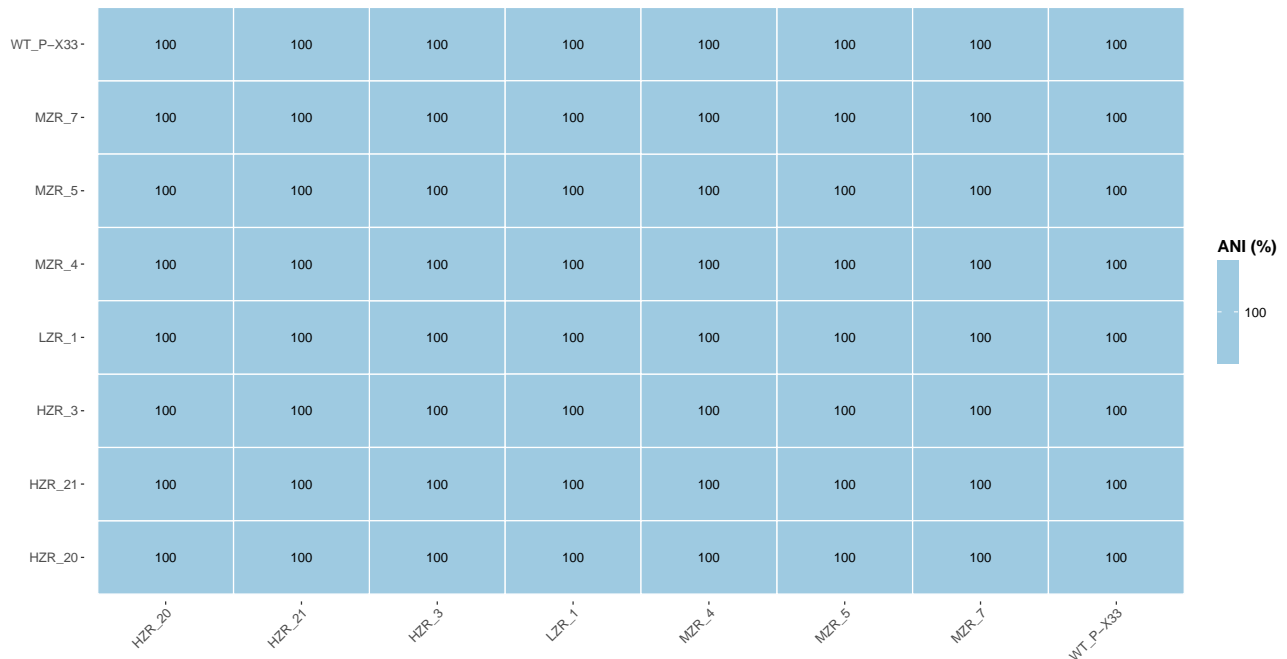

Figure 4: Average nucleotide identity. Pairwise comparison of assembled genomes based on average nucleotide identity (ANI, %).

3.5 Functional Gene Annotation

Functional gene annotation was obtained using the eggNOG database ([eggNOG 5.0](#)), which assigns orthologous groups and functional classifications based on evolutionary relationships.

This annotation process included the assignment of Enzyme Commission (EC) numbers, KEGG ortholog (KO) identifiers, KEGG pathway mapping, KEGG modules, and PFAM domain identification, enabling metabolic pathway reconstruction and functional insights. KEGG pathway annotations link genes to known metabolic, signaling, and regulatory networks, helping to infer organism-specific capabilities in processes such as carbohydrate metabolism, amino acid biosynthesis, oxidative phosphorylation, and stress response mechanisms. KEGG modules define functionally cohesive sets of genes involved in specific biological processes, such as ribosome biogenesis, glycolysis, and the TCA cycle. Additionally, KEGG reaction assignments provide insight into enzyme-catalyzed transformations, identifying key metabolic steps, substrate dependencies, and potential regulatory interactions.

Beyond metabolic reconstruction, the annotation pipeline includes KEGG BRITE hierarchical classifications, which organize genes into functional categories such as cellular processes, environmental adaptation, and signal transduction. The KEGG transporter classification (KEGG_TC) was also used to identify membrane transport proteins, shedding light on substrate uptake, efflux mechanisms, and potential antimicrobial resistance traits. Additionally, genes encoding carbohydrate-active enzymes (CAZy) were identified, providing insights into glycosyl hydrolases, transferases, and carbohydrate-binding modules involved in cell wall degradation and remodeling. BiGG reaction identifiers further enabled integration with genome-scale metabolic models, linking annotated genes to curated metabolic reconstructions used in systems biology approaches.

These annotations collectively support downstream analyses such as metabolic pathway optimization, functional enrichment studies, gene-mining for novel enzymes, and comparative genomics. Table 5 presents an overview of the extended functional annotations derived from eggNOG, summarizing key functional attributes of the predicted genes.

Table 5: Functional Annotations. This table presents functional classifications derived from the eggNOG database. **Samplename** refers to the customer-assigned sample identifier. **Locus tag** serves as the systematic identifier for each gene. **Description** offers a concise functional summary of each gene. **Gene name** denotes the known or predicted gene identifier. **EC numbers** classify enzymes, while **KEGG pathway** and **PFAM** annotations provide insights into biochemical pathways and protein domain structures. For clarity, only a subset is displayed. The complete table is available in [CMD00707_genome_stats.xlsx](#).

Samplename	Locus tag	Description	Gene	EC	KEGG pathway	PFAM
1_MZR_7	mRNA_1_MZR_7	Pfam:PA14_2	-	-	-	Flocculin,Flocculin_t3,GLEYA
2_MZR_7	mRNA_2_MZR_7	-	-	-	-	-
3_MZR_7	mRNA_3_MZR_7	Fungal specific transcription factor domain	STB4	-	-	Fungal_trans,Zn_clus
4_MZR_7	mRNA_4_MZR_7	Fungal specific transcription factor domain	STB4	-	-	Fungal_trans,Zn_clus
5_MZR_7	mRNA_5_MZR_7	Major Facilitator Superfamily	-	-	-	MFS_1
6_MZR_7	mRNA_6_MZR_7	to uniprot P40445 Saccharomyces cerevisiae YIL166c	-	-	-	MFS_1
7_MZR_7	mRNA_7_MZR_7	to uniprot Q12358 Saccharomyces cerevisiae YLL0...	-	1.14.11.17	ko00430,ko00920,m...	TauD
8_MZR_7	mRNA_8_MZR_7	-	-	-	-	-
9_MZR_7	mRNA_9_MZR_7	Superoxide dismutase	-	1.15.1.1	ko04146,ko04213,k...	Sod_Cu
10_MZR_7	mRNA_10_MZR_7	dioxygenase	-	1.14.11.17	ko00430,ko00920,m...	TauD
11_MZR_7	mRNA_11_MZR_7	protein with similarity to the allantoin perme...	-	-	-	MFS_1
12_MZR_7	mRNA_12_MZR_7	ABC transporter transmembrane region	-	3.6.3.44	ko02010,ko04976,k...	ABC_membrane,ABC_tran
13_MZR_7	mRNA_13_MZR_7	Zinc-binding dehydrogenase	-	-	-	ADH_N,ADH_zinc_N
14_MZR_7	mRNA_14_MZR_7	Permease	CAN1	-	-	AA_permease
15_MZR_7	mRNA_15_MZR_7	Belongs to the major facilitator superfamily. S...	-	-	-	Sugar_tr
16_MZR_7	mRNA_16_MZR_7	2OG-Fe(II) oxygenase superfamily	-	-	-	2OG-FelI_Oxy,DIOX_N
17_MZR_7	mRNA_17_MZR_7	Saccharomyces cerevisiae	SEO1	-	-	MFS_1
18_MZR_7	mRNA_18_MZR_7	to Saccharomyces cerevisiae YPC1 (YBR183W) and ...	YDC1	-	ko00600,ko01100,m...	Ceramidase
19_MZR_7	mRNA_19_MZR_7	to Saccharomyces cerevisiae BEM4 (YPL161C)	-	-	-	-
20_MZR_7	mRNA_20_MZR_7	Lipase (class 3)	-	-	-	Lipase_3
21_MZR_7	mRNA_21_MZR_7	to Saccharomyces cerevisiae SKY1 (YMR216C)	SKY1	2.7.11.1	-	Pkinase
22_MZR_7	mRNA_22_MZR_7	Belongs to the heat shock protein 70 family	SSE1	-	ko04141,ko04530,k...	HSP70
23_MZR_7	mRNA_23_MZR_7	Involved in the import of nuclear-targeted prot...	NPL4	-	ko04141,map04141	NPL4,UN_NPL4,zf-NPL4
24_MZR_7	mRNA_24_MZR_7	Oxidoreductase-like protein, N-terminal	-	-	-	Oxidored-like
25_MZR_7	mRNA_25_MZR_7	Splits internally a 1,3-beta-glucan molecule an...	gel2	-	-	Glyco_hydro_72
26_MZR_7	mRNA_26_MZR_7	Saccharomyces cerevisiae YPL066w	-	-	-	Arrestin_C
27_MZR_7	mRNA_27_MZR_7	Putative TOS1-like glycosyl hydrolase (DUF2401)	TOS1	-	-	DUF2401,DUF2403
28_MZR_7	mRNA_28_MZR_7	Core component of nucleosome. Nucleosomes wrap ...	HHF1	-	ko05034,ko05203,k...	CENP-T_C
29_MZR_7	mRNA_29_MZR_7	Core component of nucleosome. Nucleosomes wrap ...	HHT1	-	ko05034,ko05202,k...	Histone
30_MZR_7	mRNA_30_MZR_7	Mannosyltransferase involved in glycosylphospha...	GPI18	-	ko00563,ko01100,m...	Mannosyl_trans2
31_MZR_7	mRNA_31_MZR_7	26S proteasome non-ATPase regulatory subunit 8	RPN12	-	ko03050,ko05169,m...	CSN8_PSD8_EIF3K
32_MZR_7	mRNA_32_MZR_7	Cyclophilin type peptidyl-prolyl cis-trans isom...	CWC27	5.2.1.8	-	Pro_isomerase

3.6 BleoR and Brazzein Copy Number Evaluation

Figure 5 presents the copy number of the brazzein and BleoR genes across samples, based on BLAST alignments against the assembled genomes using the respective gene sequences as queries. These results provide insights into the relative abundance and potential genomic integration of the target genes in each sample. Supplementary Table 6 contains detailed alignment metrics, including hit counts and sequence identity values.

Figures 6 and 7 illustrate the genomic locations of BLAST hits within a representative assembly, offering spatial context for the gene distributions. These visualizations help clarify whether the genes are clustered, dispersed, or located in specific genomic regions. Additional plots for the remaining samples are shown as supplementary figures 8-20.

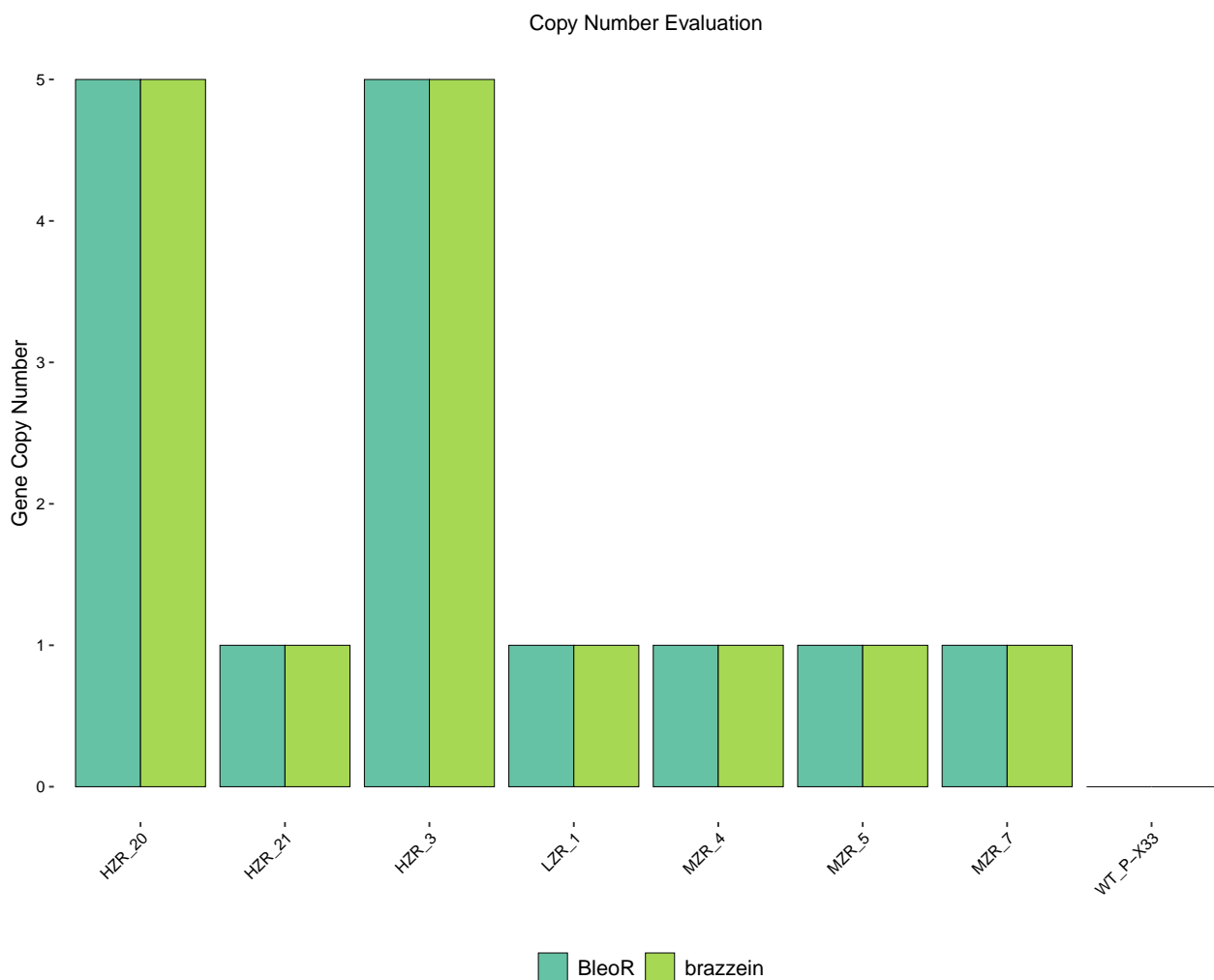

Figure 5: Gene copy number. This bar plot shows the number of detected copies for each gene (brazzein and BleoR) across different samples. Each sample is represented with side-by-side bars for each gene, allowing direct comparison of gene presence and abundance. The sample WT_P-X33 is included as a negative control with zero counts for both genes.

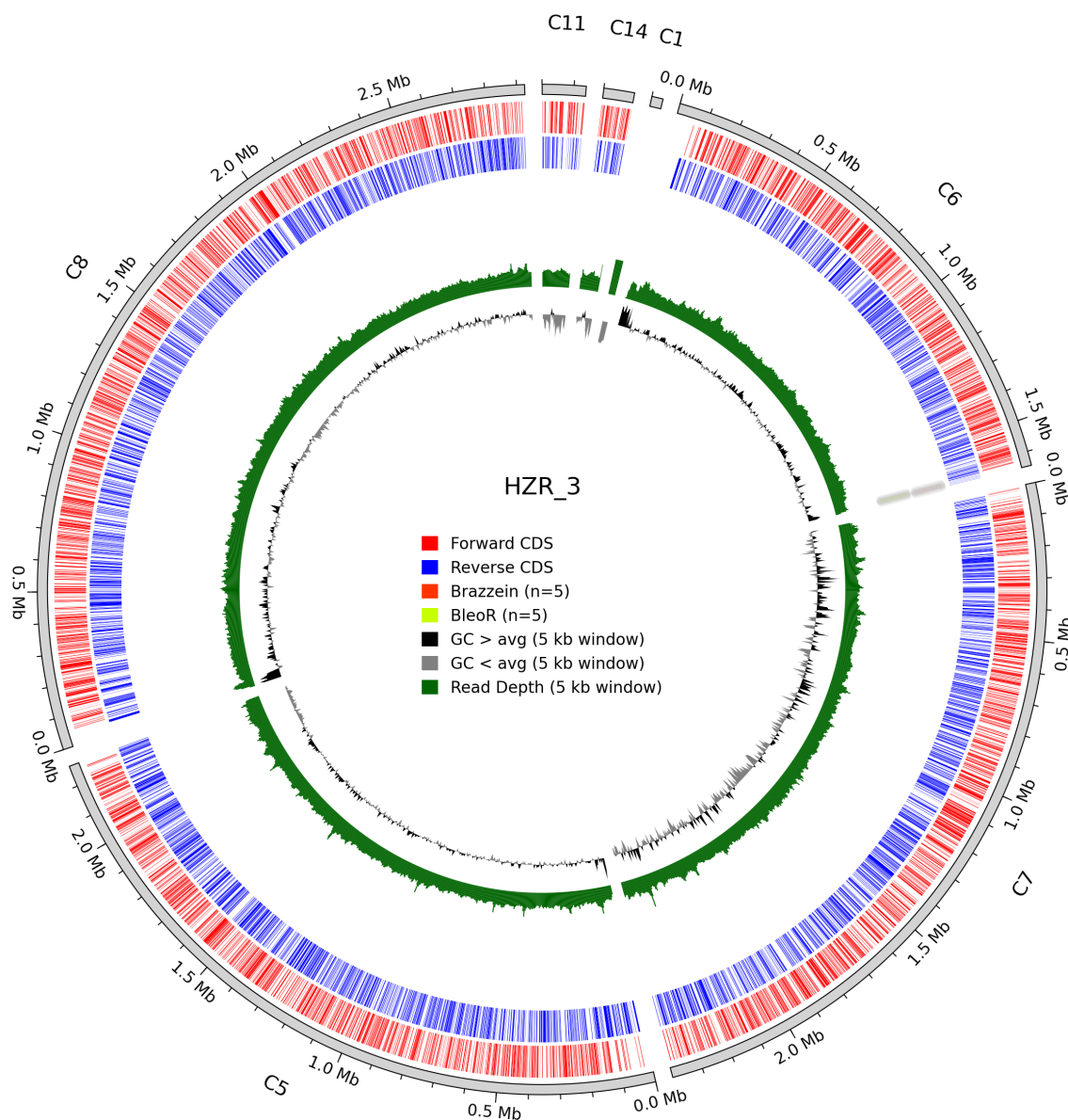

Figure 6: Circular genome visualization. This plot displays a circular representation of an assembled genome. The outermost track shows coding sequences (CDS), followed by tracks marking the genomic locations of BLAST hits for the *brazzein* and *BleoR* query sequences, providing insight into their distribution across the genome. A depth track illustrates sequencing coverage, capped at 500× to enhance readability. The innermost track highlight the GC content: regions above and below the average GC content are highlighted to indicate compositional variation.

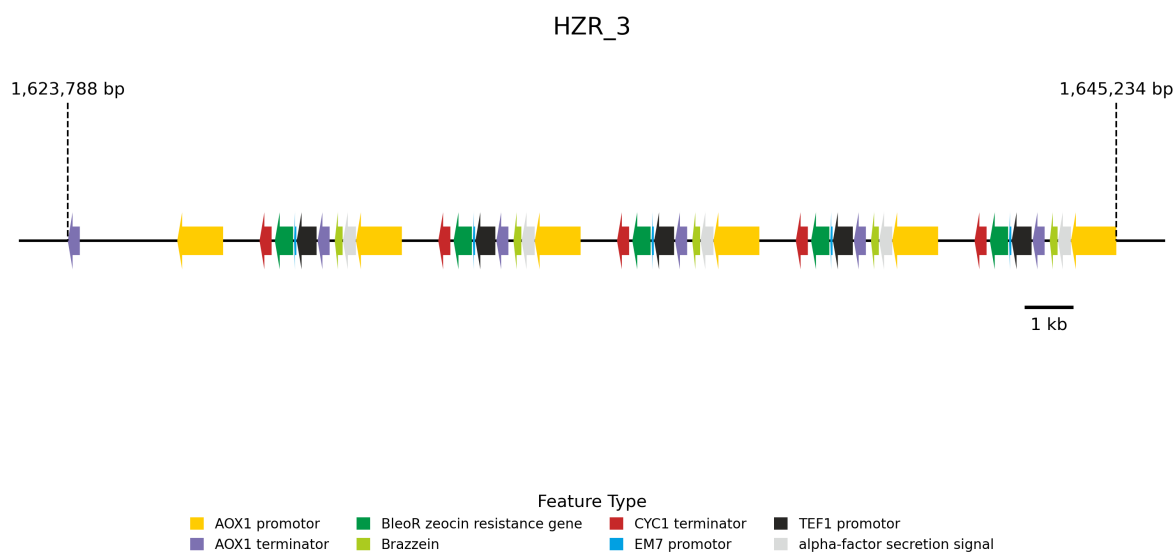

Figure 7: Zoomed-in view of BLAST hit regions. This figure highlights a specific genomic region from the circular genome plot, zoomed to show the precise locations of BLAST hits for the brazzein and BleoR query sequences.

4 Materials and methods

4.1 DNA extraction and quality control

High molecular weight DNA was extracted using the Monarch® HMW DNA Extraction Kit (NEB, USA), following a yeast-specific protocol. The procedure included enzymatic lysis of the yeast cell wall with Zymolase (5 U/μl) in Spheroplast Buffer composed of 1 M sorbitol, 1× PBS (pH 7.4), and 0.1 M EDTA. Subsequent digestion steps were performed with Proteinase K and RNase A to remove proteins and RNA contaminants.

DNA concentration was measured using the Qubit dsDNA HS Assay Kit (Thermo Fisher Scientific, USA), while purity was assessed with the NanoDrop One spectrophotometer (Thermo Fisher Scientific, USA). Fragment size distribution was evaluated using the Agilent TapeStation 4200 system with Genomic DNA ScreenTapes (Agilent, USA).

4.2 DNA sequencing

A barcoded SQK-RBK114.96 DNA library was prepared following the manufacturer's protocol with minor modifications (Oxford Nanopore Technologies, Oxford, United Kingdom). The barcoded library was then loaded onto a primed FLO-PRO114M (R10.4.1) flow cell and sequenced using a PromethION P24 device running MinKNOW Release 25.05.12. Signal data were basecalled with Dorado basecall server v. 7.9.8 (Oxford Nanopore Technologies, Oxford, United Kingdom) utilizing the super-accurate basecalling algorithm (dna_r10.4.1_e8.2_400bps_5khz_sup.cfg). Residual adapters were removed with Porechop v. 0.2.4, followed by read length filtering (-l 1000) and quality filtering (-q 20) using Nanoq v. 0.10.0 [6]. Basic sequencing statistics were obtained using Nanoq v. 0.10.0 [6].

4.3 De novo assembly

De novo assemblies were generated using Flye v. 2.9.4 [7–9] with the parameters --meta and --extra-params min_read_cov_cutoff=10. The draft assemblies were subsequently polished once with Medaka v. 2.0.1 (Oxford Nanopore Technologies, Oxford, United Kingdom). Assembly graphs were generated and inspected using Bandage v. 0.8.1 [10]. Contigs smaller than 1000 bp were removed using Seqkit v. 2.8.2. Contigs with low mapping quality (< 30) and low coverage (< 10) were identified using Minimap2 v. 2.29 and Samtools v. 1.21 stats and removed using GNU Awk v. 5.3.0. Quast v. 5.2.0 was used to obtain basic assembly statistics [11]. Genome completeness was evaluated using Busco v. 5.8.3, executed in genome auto-lineage-euk mode [3].

4.4 Gene annotation

Genomes were *ab initio* gene-annotated using Braker3 v. 3.0.8 with GeneMark-ES [12], applying the --fungus flag for fungal isolates. Transcript and protein sequences were extracted using Gffread v. 0.12.7 [13], and identifiers were suffixed with isolate-specific names. Predicted proteins were combined to generate a custom protein database. Functional annotation of protein sequences was performed using eggNOG-mapper v2.1.12 [14], which assigns orthology and functional information based on the eggNOG 5.0 database [15]. The annotation process utilized for sequence homology searches against the eggNOG orthologous groups [16]. The protein sequences were annotated with eggNOG-mapper with the parameters --sensmode more-sensitive, --tax_scope auto and --go_evidence all.

4.5 Classification

Internal transcribed spacer (ITS) regions, specifically ITS1 and ITS2, were delineated from genome assemblies using a custom Python-based extraction pipeline configured with region-specific parameters. Subsequent taxonomic classification of the extracted ITS sequences was conducted using Constax v. 2.0.20 [17], employing the UNITE reference database (release 19.02.2025) [18]. Constax v. 2.0.20 was executed with the --blast option and a minimum confidence threshold of 0.8 to ensure robust taxonomic assignments. Resulting classifications were parsed from syntax output files to identify unique taxonomic lineages.

Ribosomal RNA (rRNA) gene sequences were identified using Barrnap v. 0.9 with the --kingdom euk parameter to target eukaryotic rDNA regions. The extracted sequences were subsequently aligned and taxonomically annotated using SINA v. 1.7.2 [19] against the SILVA SSURef NR99 database (release 138.1) [20], enabling high-resolution phylogenetic placement based on small subunit rRNA gene sequences.

4.6 BLAST

To identify homologous regions, each assembled genome was formatted as a nucleotide BLAST database using the makeblastdb utility from BLAST+ version 2.16.0 with the command `makeblastdb -in genome.fasta -dbtype nucl -out genome_blastdb`. The query dataset consisted of customer-supplied gene sequences for *brazzein* and *BleoR*, which were aligned against each genome database using `blastn`. The alignment was performed with the command `blastn -query genome.fasta -db genome_blastdb -out genome_blastn_results.out -outfmt "6 qseqid sseqid pident nident mismatch gaps gapopen qstart qend sstart send qseq sseq evalue bitscore qlen slen" -evalue 0.1 -max_target_seqs 50`.

The output format was customized to include detailed alignment metrics such as sequence identity, alignment length, mismatches, gaps, query and subject coordinates, raw sequences, e-value, bitscore, and sequence lengths. This configuration enabled comprehensive downstream analysis of alignment quality and genomic context.

5 Supplementary information

5.1 BLAST Results

Table 6: BLASTn Results. This table summarizes sequence alignment results from BLASTn analysis. **Source** indicates the sample identifier. **qseqid** is the query sequence name, and **sseqid** is the subject sequence name. **pident** shows the percentage of identical matches. **Length**, **Mismatch**, and **Gapopen** describe alignment metrics. **qstart** and **qend** mark the query alignment positions, while **sstart** and **send** mark the subject alignment positions. **E-value** and **Bitscore** provide statistical significance and alignment score. For clarity, only a subset is displayed. The complete table is available in CMD00707_genome_stats.xlsx.

Source	Query ID	Subject ID	Identity %	Length	Mismatch	Gap Open	Query Start	Query End	Subject Start	Subject End	E-value	Bitscore
HZR_20	brazzein	contig_12_299x_lin_1644568bp_58.5	100	162	0	0	1	162	1628743	1628582	0	300
HZR_20	brazzein	contig_12_299x_lin_1644568bp_58.5	100	162	0	0	1	162	1632399	1632238	0	300
HZR_20	brazzein	contig_12_299x_lin_1644568bp_58.5	100	162	0	0	1	162	1636055	1635894	0	300
HZR_20	brazzein	contig_12_299x_lin_1644568bp_58.5	100	162	0	0	1	162	1639711	1639550	0	300
HZR_20	brazzein	contig_12_299x_lin_1644568bp_58.5	100	162	0	0	1	162	1643367	1643206	0	300
HZR_20	BleoR	contig_12_299x_lin_1644568bp_58.5	100	375	0	0	1	375	1627731	1627357	0	693
HZR_20	BleoR	contig_12_299x_lin_1644568bp_58.5	100	375	0	0	1	375	1631387	1631013	0	693
HZR_20	BleoR	contig_12_299x_lin_1644568bp_58.5	100	375	0	0	1	375	1635043	1634669	0	693
HZR_20	BleoR	contig_12_299x_lin_1644568bp_58.5	100	375	0	0	1	375	1638699	1638325	0	693
HZR_20	BleoR	contig_12_299x_lin_1644568bp_58.5	100	375	0	0	1	375	1642355	1641981	0	693
HZR_21	brazzein	contig_11_164x_lin_1725378bp_58.5	100	162	0	0	1	162	1626926	1626765	0	300
HZR_21	BleoR	contig_11_164x_lin_1725378bp_58.5	100	375	0	0	1	375	1625914	1625540	0	693
HZR_3	brazzein	contig_6_157x_lin_1645234bp_58.4	100	162	0	0	1	162	1629409	1629248	0	300
HZR_3	brazzein	contig_6_157x_lin_1645234bp_58.4	100	162	0	0	1	162	1633065	1632904	0	300
HZR_3	brazzein	contig_6_157x_lin_1645234bp_58.4	100	162	0	0	1	162	1636721	1636560	0	300
HZR_3	brazzein	contig_6_157x_lin_1645234bp_58.4	100	162	0	0	1	162	1640377	1640216	0	300
HZR_3	brazzein	contig_6_157x_lin_1645234bp_58.4	100	162	0	0	1	162	1644033	1643872	0	300
HZR_3	BleoR	contig_6_157x_lin_1645234bp_58.4	100	375	0	0	1	375	1628397	1628023	0	693
HZR_3	BleoR	contig_6_157x_lin_1645234bp_58.4	100	375	0	0	1	375	1632053	1631679	0	693
HZR_3	BleoR	contig_6_157x_lin_1645234bp_58.4	100	375	0	0	1	375	1635709	1635335	0	693
HZR_3	BleoR	contig_6_157x_lin_1645234bp_58.4	100	375	0	0	1	375	1639365	1638991	0	693
HZR_3	BleoR	contig_6_157x_lin_1645234bp_58.4	100	375	0	0	1	375	1643021	1642647	0	693
LZR_1	brazzein	contig_11_151x_lin_2400598bp_60	100	162	0	0	1	162	490316	490155	0	300
LZR_1	BleoR	contig_11_151x_lin_2400598bp_60	100	375	0	0	1	375	489304	488930	0	693
MZR_4	brazzein	contig_30_279x_lin_1704683bp_59.4	100	162	0	0	1	162	1606232	1606071	0	300
MZR_4	BleoR	contig_30_279x_lin_1704683bp_59.4	100	375	0	0	1	375	1605220	1604846	0	693
MZR_5	brazzein	contig_11_162x_lin_1725388bp_58	100	162	0	0	1	162	1626935	1626774	0	300
MZR_5	BleoR	contig_11_162x_lin_1725388bp_58	100	375	0	0	1	375	1625923	1625549	0	693
MZR_7	brazzein	contig_11_194x_lin_1718893bp_58.5	100	162	0	0	1	162	1620442	1620281	0	300
MZR_7	BleoR	contig_11_194x_lin_1718893bp_58.5	100	375	0	0	1	375	1619430	1619056	0	693

5.2 Circos Plots

5.2.1 HZR_20

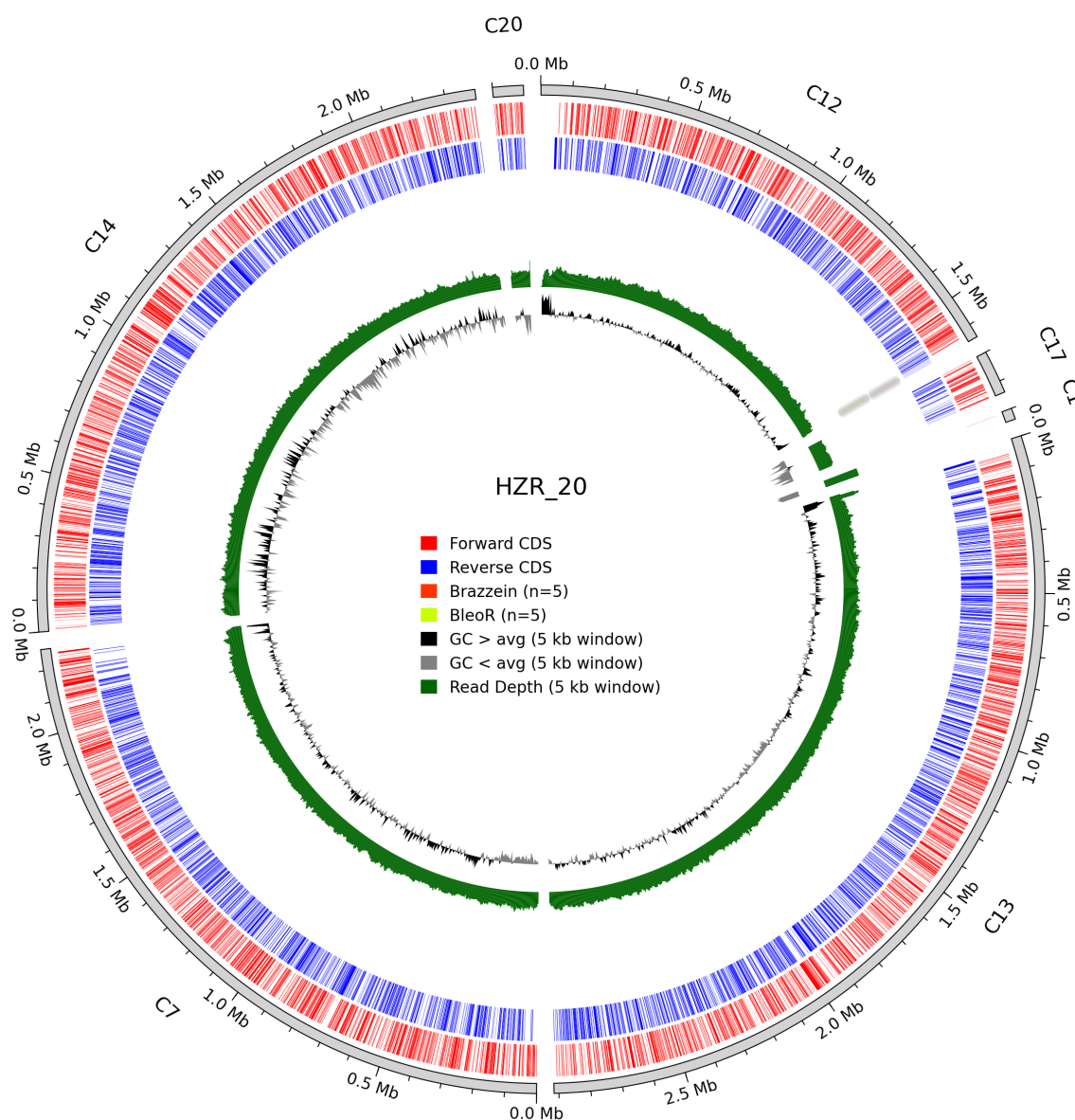

Figure 8: Circular genome visualization. This plot displays a circular representation of an assembled genome. The outermost track shows coding sequences (CDS), followed by tracks marking the genomic locations of BLAST hits for the brazzein and BleoR query sequences, providing insight into their distribution across the genome. A depth track illustrates sequencing coverage, capped at 500× to enhance readability. The innermost track highlight the GC content: regions above and below the average GC content are highlighted to indicate compositional variation.

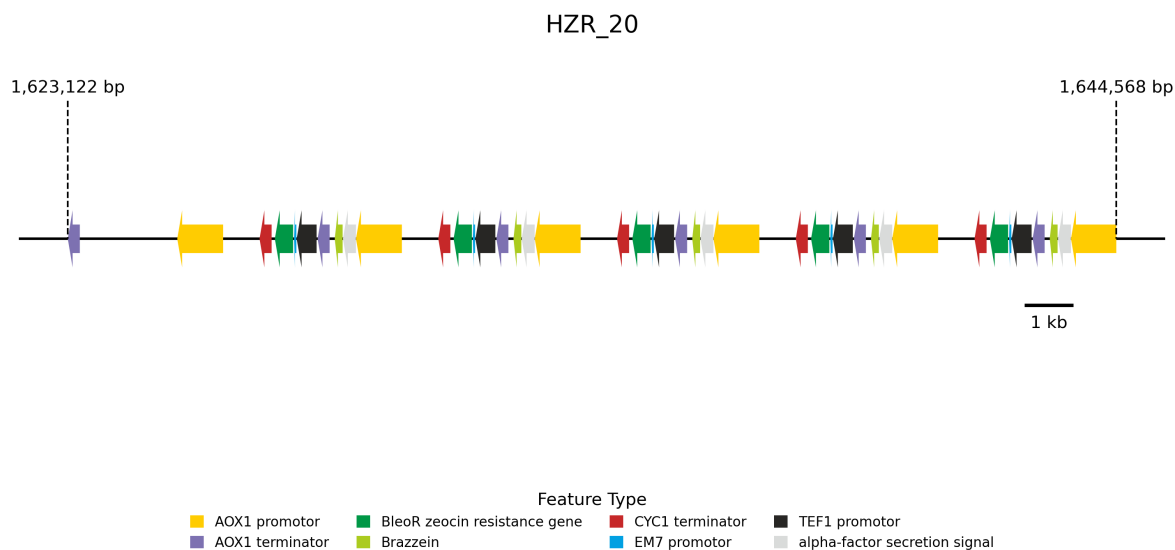

Figure 9: Zoomed-in view of BLAST hit regions. This figure highlights a specific genomic region from the circular genome plot, zoomed to show the precise locations of BLAST hits for the brazzein and BleoR query sequences.

5.2.2 HZR_21

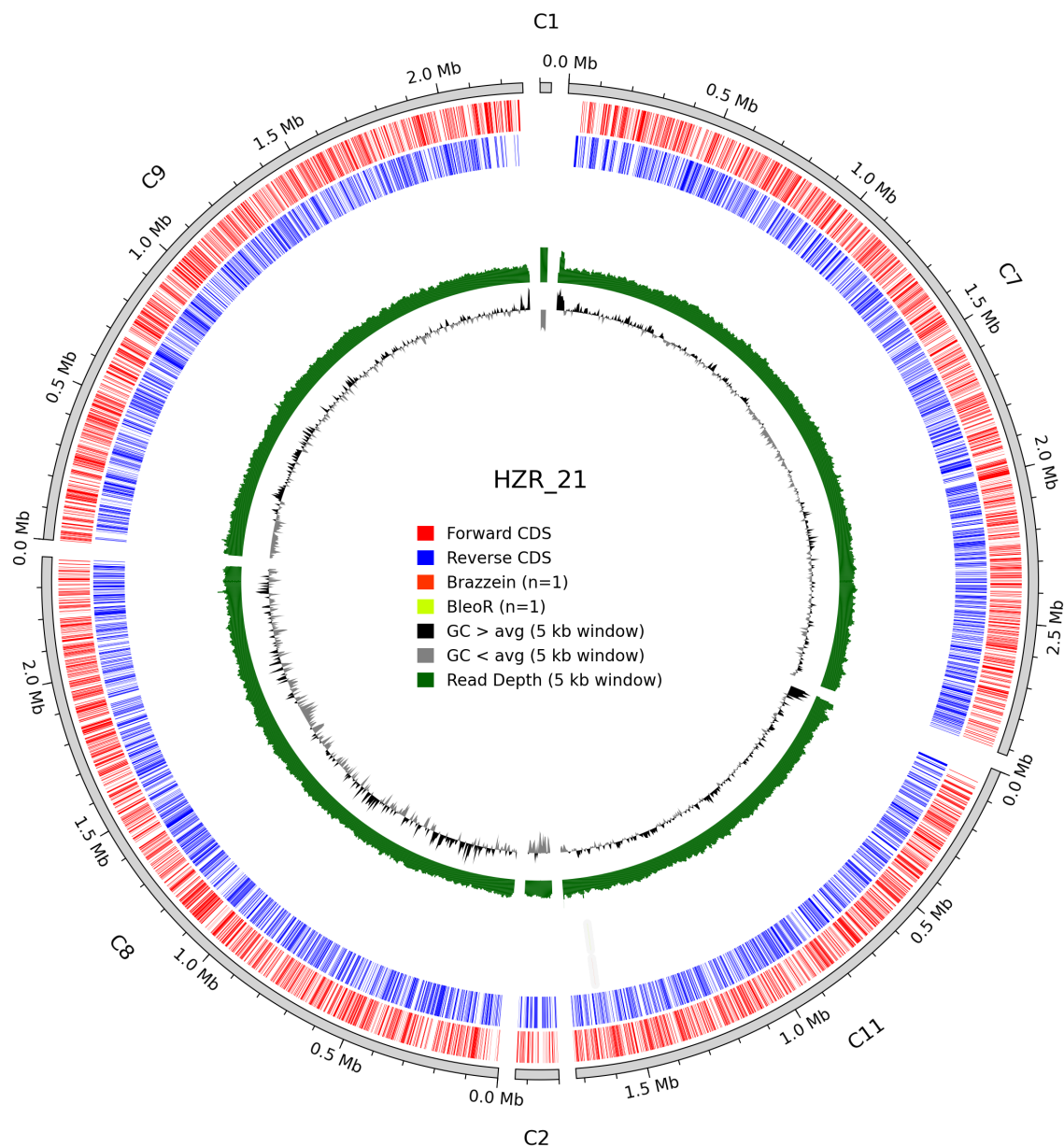

Figure 10: Circular genome visualization. This plot displays a circular representation of an assembled genome. The outermost track shows coding sequences (CDS), followed by tracks marking the genomic locations of BLAST hits for the *brazzein* and *BleoR* query sequences, providing insight into their distribution across the genome. A depth track illustrates sequencing coverage, capped at 500× to enhance readability. The innermost track highlight the GC content: regions above and below the average GC content are highlighted to indicate compositional variation.

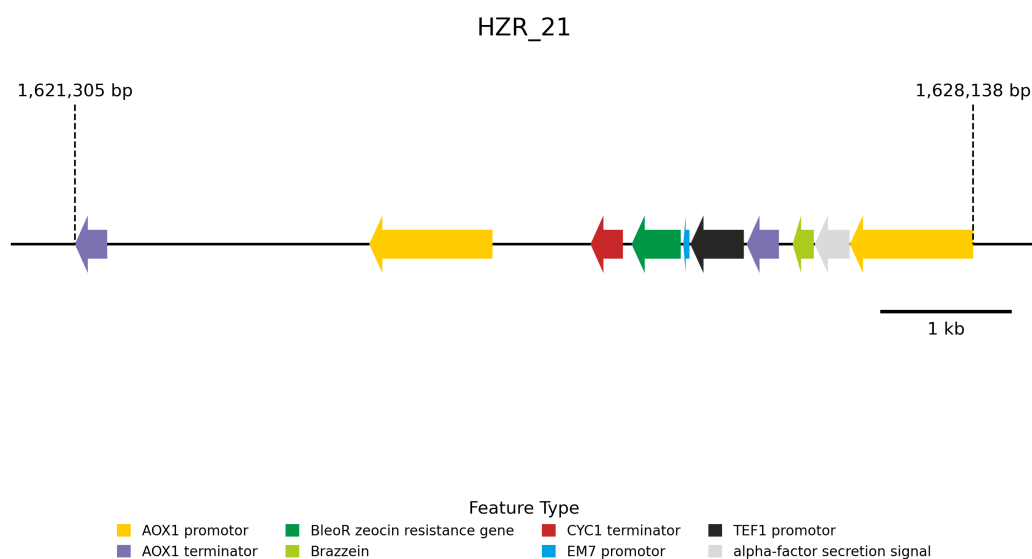

Figure 11: Zoomed-in view of BLAST hit regions. This figure highlights a specific genomic region from the circular genome plot, zoomed to show the precise locations of BLAST hits for the *brazzein* and *BleoR* query sequences.

5.2.3 LZR_1

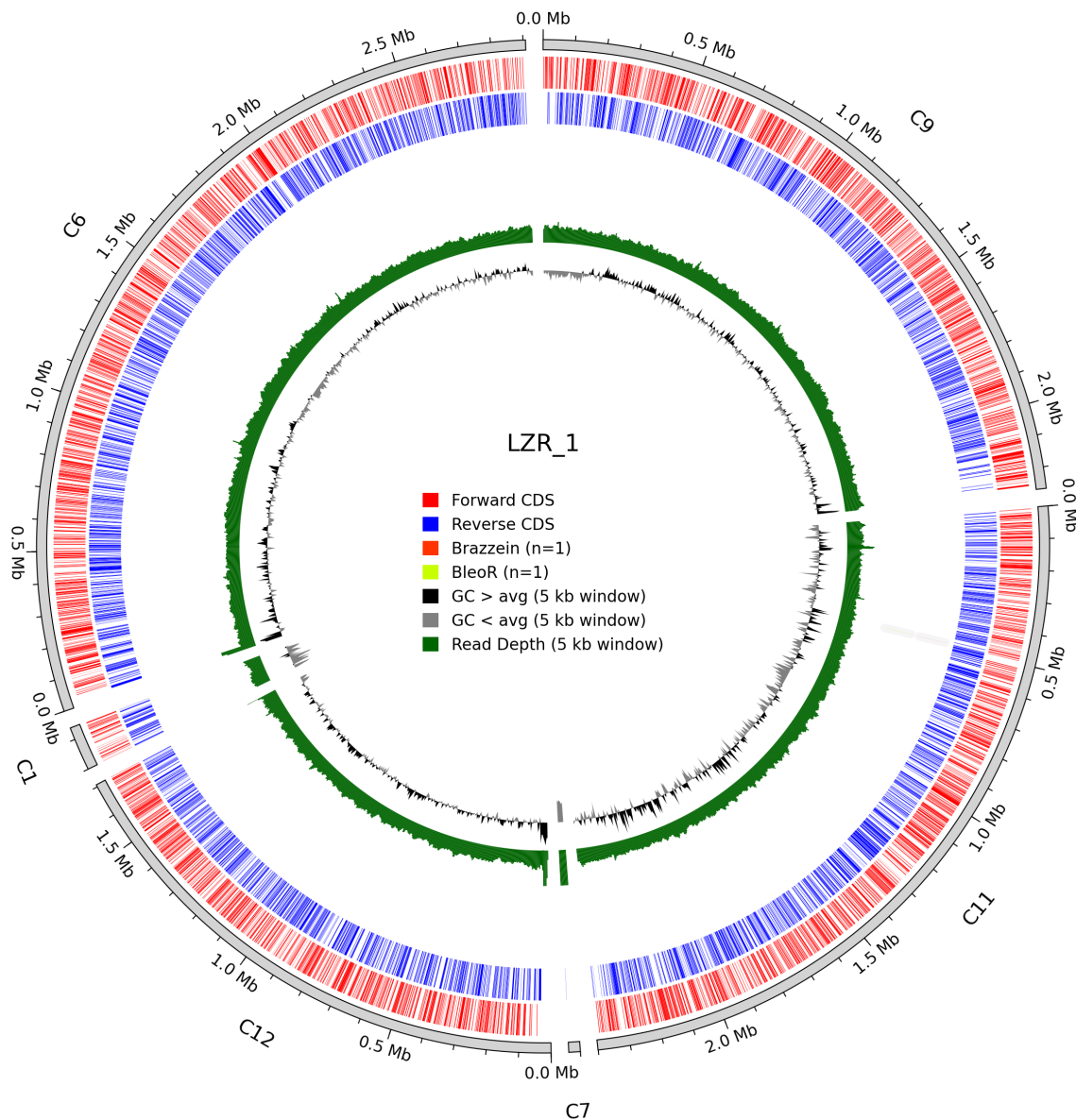

Figure 12: Circular genome visualization. This plot displays a circular representation of an assembled genome. The outermost track shows coding sequences (CDS), followed by tracks marking the genomic locations of BLAST hits for the *brazzein* and *BleoR* query sequences, providing insight into their distribution across the genome. A depth track illustrates sequencing coverage, capped at 500 $\times$ to enhance readability. The innermost track highlight the GC content: regions above and below the average GC content are highlighted to indicate compositional variation.

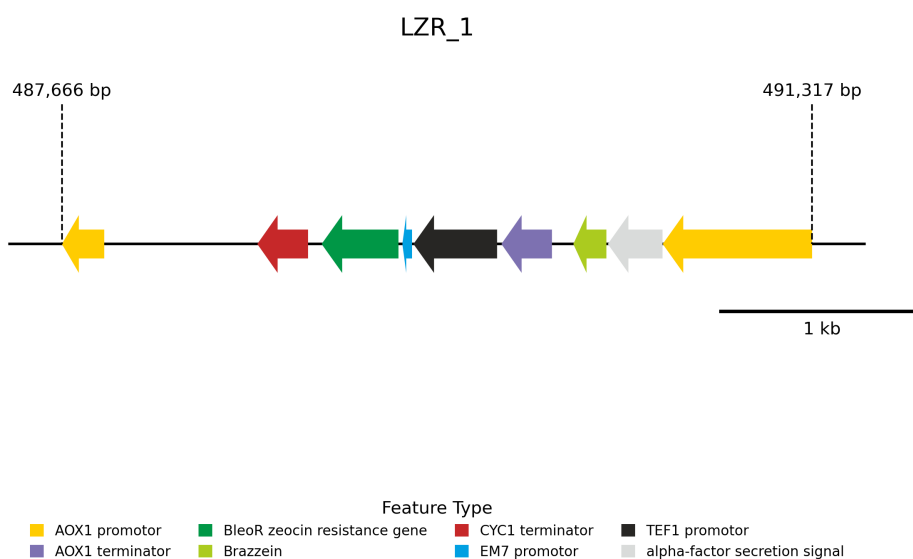

Figure 13: Zoomed-in view of BLAST hit regions. This figure highlights a specific genomic region from the circular genome plot, zoomed to show the precise locations of BLAST hits for the *brazzein* and *BleoR* query sequences.

5.2.4 MZR_4

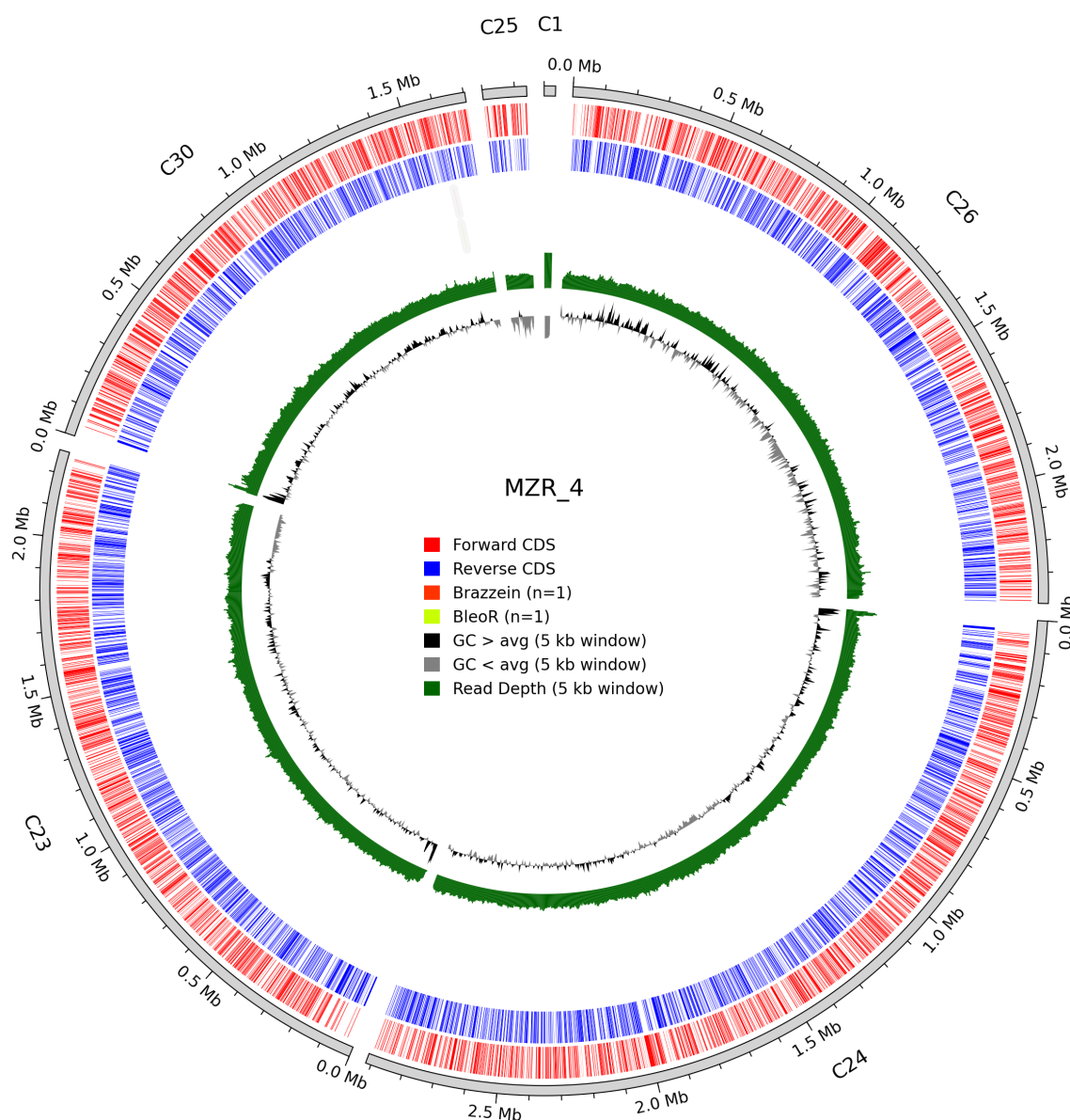

Figure 14: Circular genome visualization. This plot displays a circular representation of an assembled genome. The outermost track shows coding sequences (CDS), followed by tracks marking the genomic locations of BLAST hits for the brazzein and BleoR query sequences, providing insight into their distribution across the genome. A depth track illustrates sequencing coverage, capped at 500× to enhance readability. The innermost track highlight the GC content: regions above and below the average GC content are highlighted to indicate compositional variation.

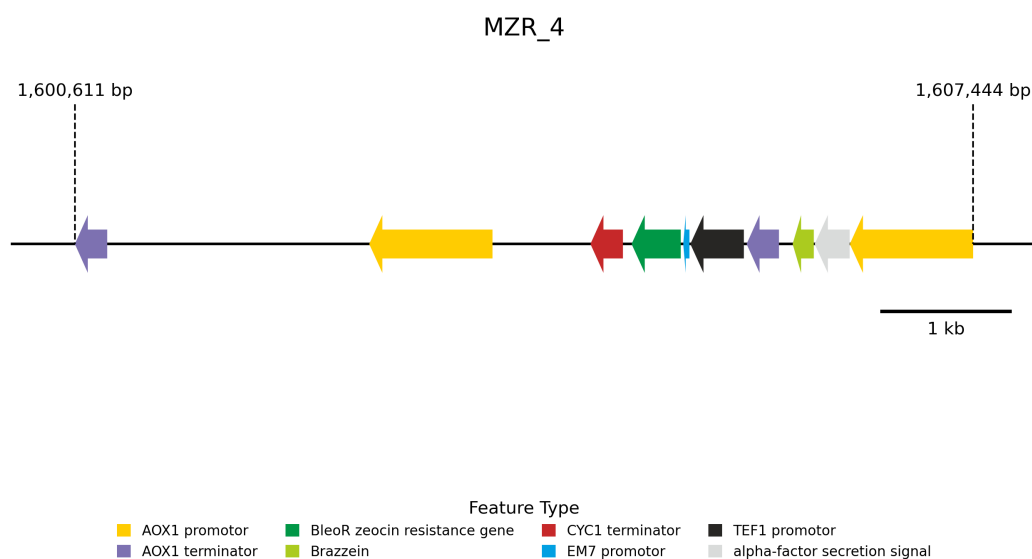

Figure 15: Zoomed-in view of BLAST hit regions. This figure highlights a specific genomic region from the circular genome plot, zoomed to show the precise locations of BLAST hits for the *brazzein* and *BleoR* query sequences.

5.2.5 MZR_5

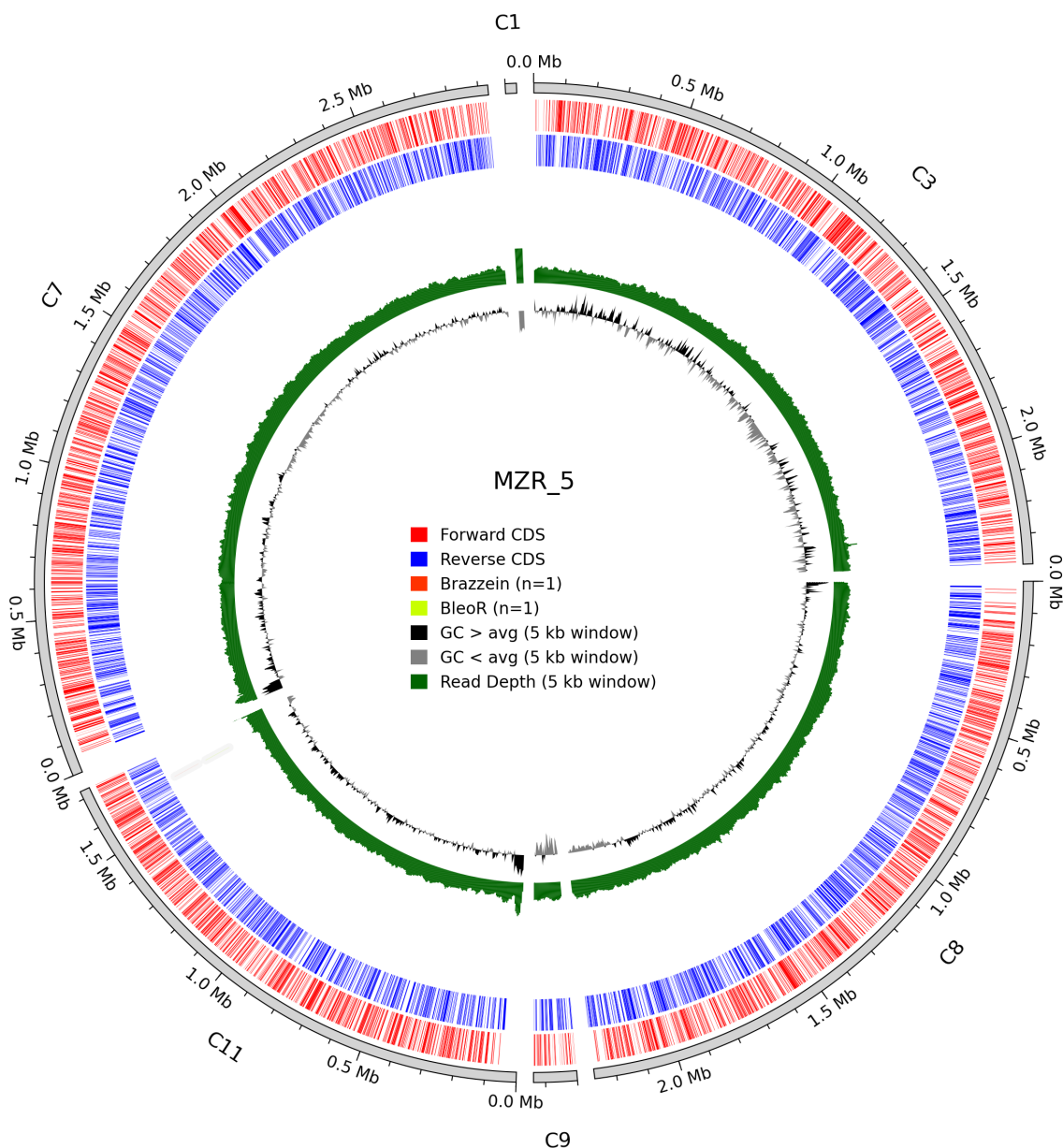

Figure 16: Circular genome visualization. This plot displays a circular representation of an assembled genome. The outermost track shows coding sequences (CDS), followed by tracks marking the genomic locations of BLAST hits for the *brazzein* and *BleoR* query sequences, providing insight into their distribution across the genome. A depth track illustrates sequencing coverage, capped at 500× to enhance readability. The innermost track highlight the GC content: regions above and below the average GC content are highlighted to indicate compositional variation.

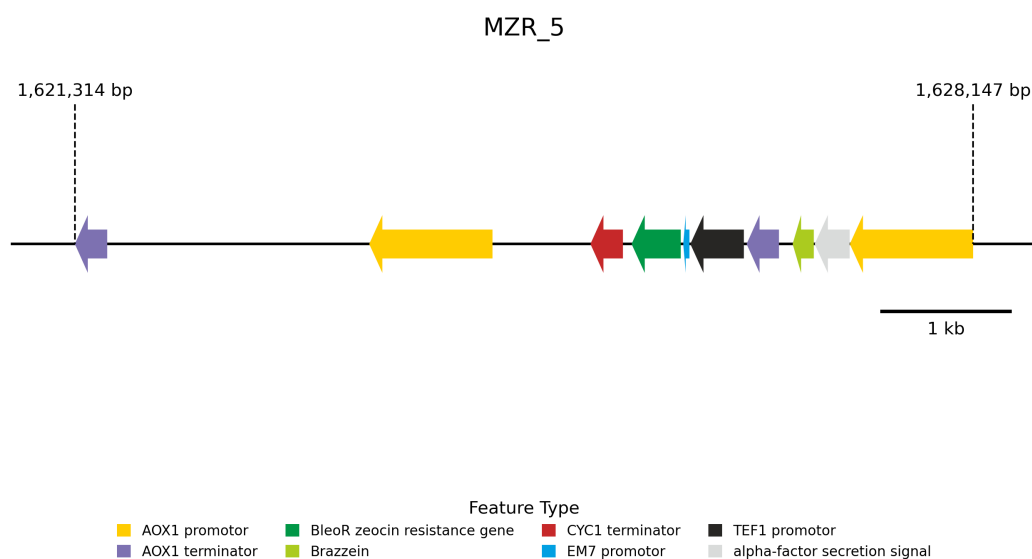

Figure 17: Zoomed-in view of BLAST hit regions. This figure highlights a specific genomic region from the circular genome plot, zoomed to show the precise locations of BLAST hits for the *brazzein* and *BleoR* query sequences.

5.2.6 MZR_7

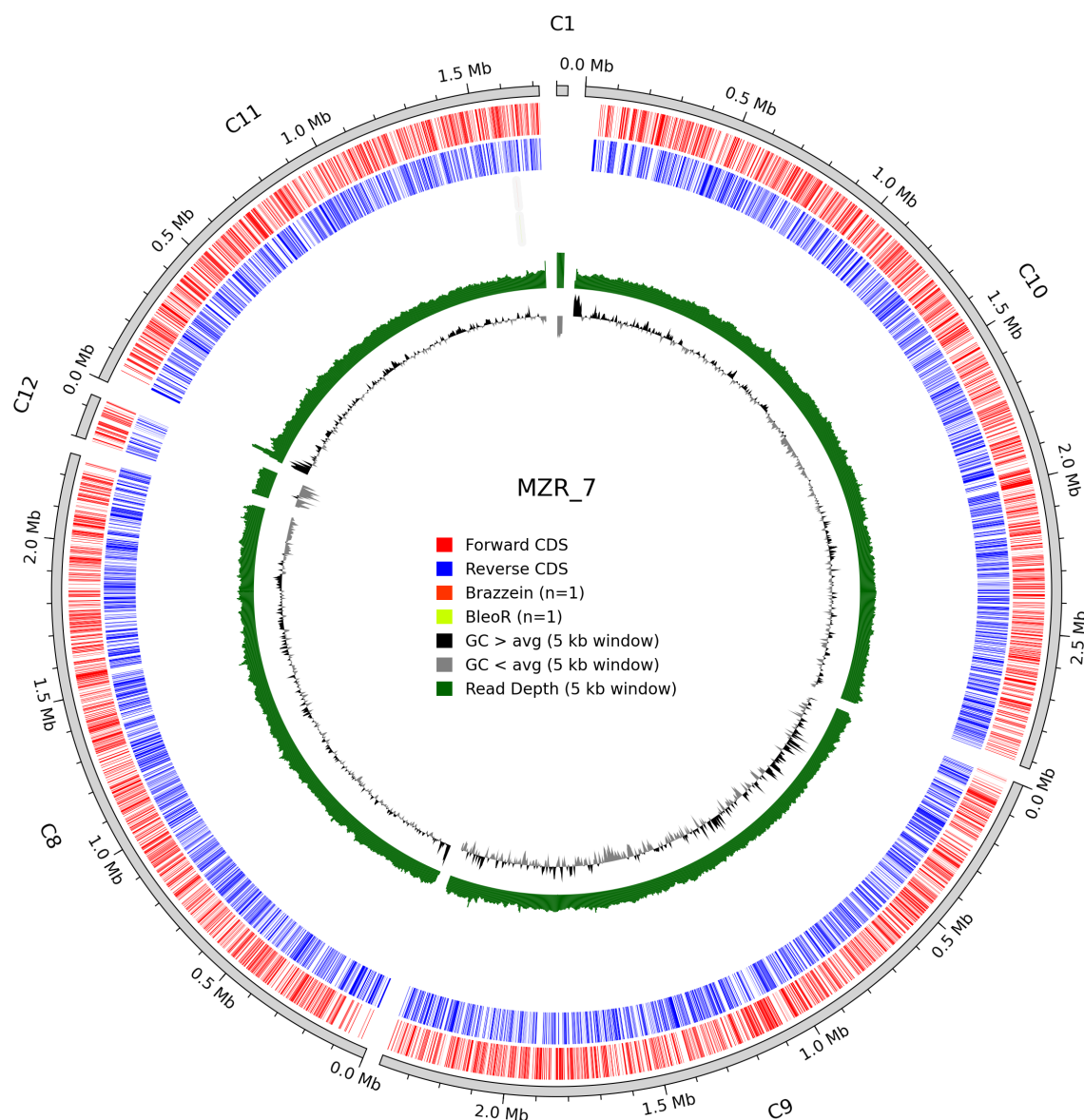

Figure 18: Circular genome visualization. This plot displays a circular representation of an assembled genome. The outermost track shows coding sequences (CDS), followed by tracks marking the genomic locations of BLAST hits for the brazzein and BleoR query sequences, providing insight into their distribution across the genome. A depth track illustrates sequencing coverage, capped at 500× to enhance readability. The innermost track highlight the GC content: regions above and below the average GC content are highlighted to indicate compositional variation.

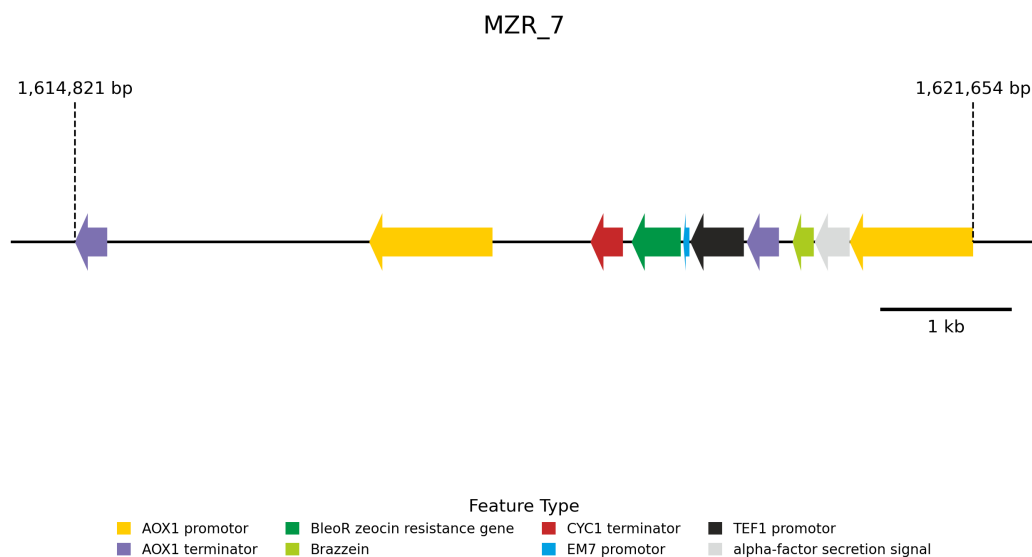

Figure 19: Zoomed-in view of BLAST hit regions. This figure highlights a specific genomic region from the circular genome plot, zoomed to show the precise locations of BLAST hits for the *brazzein* and *BleoR* query sequences.

5.2.7 WT_P-X33

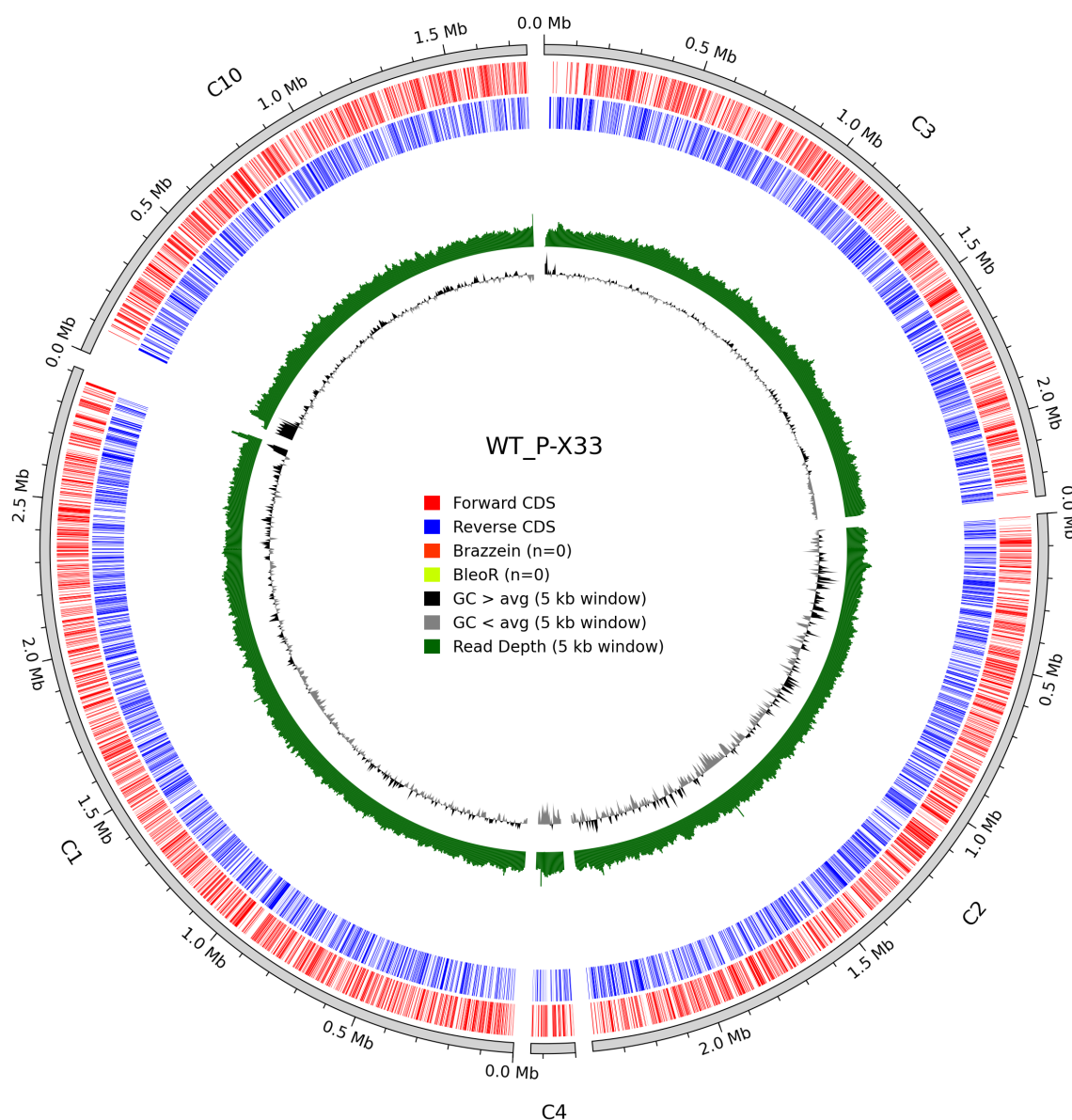

Figure 20: Circular genome visualization. This plot displays a circular representation of an assembled genome. The outermost track shows coding sequences (CDS), followed by tracks marking the genomic locations of BLAST hits for the brazzein and BleoR query sequences, providing insight into their distribution across the genome. A depth track illustrates sequencing coverage, capped at 500× to enhance readability. The innermost track highlight the GC content: regions above and below the average GC content are highlighted to indicate compositional variation.

5.3 Raw assembly contigs

Table 7: List of nonfiltered contigs across all samples. Each contig is labeled with key attributes in the format: *contig_depth_topology_plasmid_length_mapping-quality*. The mapping quality score, output by minimap2 and samtools, indicates the confidence of correct read placement, with higher scores (e.g., ≥ 30) representing high-confidence mappings. Lower scores suggest ambiguous mapping, potentially due to repetitive regions or multiple alignment locations.

samplename	Contig
MZR_5	contig_3_161x_lin_2396772bp_60
MZR_5	contig_8_165x_lin_2268880bp_59.8
MZR_5	contig_9_186x_lin_137991bp_58.5
MZR_5	contig_11_162x_lin_1725388bp_58
MZR_5	contig_7_164x_lin_2931336bp_59
MZR_5	contig_1_6293x_circ_35687bp_59.9
HZR_3	contig_11_174x_lin_137991bp_57.9
HZR_3	contig_14_167x_lin_97238bp_60
HZR_3	contig_1_6731x_circ_35688bp_60
HZR_3	contig_6_157x_lin_1645234bp_58.4
HZR_3	contig_7_154x_lin_2396489bp_60
HZR_3	contig_5_157x_lin_2266596bp_59.9
HZR_3	contig_8_158x_lin_2925239bp_59.3
HZR_21	contig_1_5502x_circ_35687bp_59.9
HZR_21	contig_7_169x_lin_2920891bp_59
HZR_21	contig_11_164x_lin_1725378bp_58.5
HZR_21	contig_2_178x_lin_137991bp_58.4
HZR_21	contig_8_167x_lin_2396656bp_60
HZR_21	contig_9_169x_lin_2266691bp_59.9
HZR_20	contig_12_299x_lin_1644568bp_58.5
HZR_20	contig_17_313x_lin_137992bp_57.9
HZR_20	contig_18_535x_lin_4778bp_0.28
HZR_20	contig_3_20x_lin_1080bp_0.6
HZR_20	contig_1_11990x_circ_35689bp_57.9
HZR_20	contig_13_303x_lin_2915119bp_59.3
HZR_20	contig_7_306x_lin_2266796bp_59.9
HZR_20	contig_14_303x_lin_2396338bp_60
HZR_20	contig_20_328x_lin_97241bp_59.9
WT_P-X33	contig_3_88x_lin_2271904bp_59.9
WT_P-X33	contig_2_88x_lin_2396484bp_60
WT_P-X33	contig_6_2980x_lin_288bp_59.7
WT_P-X33	contig_4_103x_lin_137993bp_58.4
WT_P-X33	contig_1_87x_lin_2916224bp_54.7
WT_P-X33	contig_10_85x_lin_1753055bp_58.8
WT_P-X33	contig_7_3001x_lin_527bp_59.6
WT_P-X33	contig_5_3241x_lin_522bp_58.7
MZR_7	contig_1_8087x_circ_35339bp_59
MZR_7	contig_10_196x_lin_2924289bp_59.4
MZR_7	contig_9_191x_lin_2396565bp_60
MZR_7	contig_8_195x_lin_2269999bp_59.9

5.4 Quality-filtered contigs

Table 8: List of filtered contigs across all samples. Each contig is labeled with key attributes in the format: *contig_depth_topology_plasmid_length_mapping-quality*. The mapping quality score, output by minimap2 and samtools, indicates the confidence of correct read placement, with higher scores (e.g., ≥ 30) representing high-confidence mappings. Lower scores suggest ambiguous mapping, potentially due to repetitive regions or multiple alignment locations.

samplename	Contig
HZR_20	contig_12_299x_lin_1644568bp_58.5
HZR_20	contig_17_313x_lin_137992bp_57.9
HZR_20	contig_1_11990x_circ_35689bp_57.9
HZR_20	contig_13_303x_lin_2915119bp_59.3
HZR_20	contig_7_306x_lin_2266796bp_59.9
HZR_20	contig_14_303x_lin_2396338bp_60
HZR_20	contig_20_328x_lin_97241bp_59.9
HZR_21	contig_1_5502x_circ_35687bp_59.9
HZR_21	contig_7_169x_lin_2920891bp_59
HZR_21	contig_11_164x_lin_1725378bp_58.5
HZR_21	contig_2_178x_lin_137991bp_58.4
HZR_21	contig_8_167x_lin_2396656bp_60
HZR_21	contig_9_169x_lin_2266691bp_59.9
HZR_3	contig_11_174x_lin_137991bp_57.9
HZR_3	contig_14_167x_lin_97238bp_60
HZR_3	contig_1_6731x_circ_35688bp_60
HZR_3	contig_6_157x_lin_1645234bp_58.4
HZR_3	contig_7_154x_lin_2396489bp_60
HZR_3	contig_5_157x_lin_2266596bp_59.9
HZR_3	contig_8_158x_lin_2925239bp_59.3
LZR_1	contig_9_152x_lin_2266591bp_59.8
LZR_1	contig_11_151x_lin_2400598bp_60
LZR_1	contig_7_555x_circ_35688bp_38.6
LZR_1	contig_12_151x_lin_1706776bp_58.8
LZR_1	contig_1_168x_lin_137991bp_58
LZR_1	contig_6_151x_lin_2907667bp_58.9
MZR_4	contig_1_13780x_circ_35688bp_59.2
MZR_4	contig_26_280x_lin_2396856bp_59.9
MZR_4	contig_24_278x_lin_2917386bp_59.3
MZR_4	contig_23_279x_lin_2270661bp_59.9
MZR_4	contig_30_279x_lin_1704683bp_59.4
MZR_4	contig_25_281x_lin_137992bp_57.8
MZR_5	contig_3_161x_lin_2396772bp_60
MZR_5	contig_8_165x_lin_2268880bp_59.8
MZR_5	contig_9_186x_lin_137991bp_58.5
MZR_5	contig_11_162x_lin_1725388bp_58
MZR_5	contig_7_164x_lin_2931336bp_59
MZR_5	contig_1_6293x_circ_35687bp_59.9
MZR_7	contig_1_8087x_circ_35339bp_59
MZR_7	contig_10_196x_lin_2924289bp_59.4
MZR_7	contig_9_191x_lin_2396565bp_60
MZR_7	contig_8_195x_lin_2269999bp_59.9
MZR_7	contig_12_214x_lin_137992bp_58.2
MZR_7	contig_11_194x_lin_1718893bp_58.5
WT_P-X33	contig_3_88x_lin_2271904bp_59.9
WT_P-X33	contig_2_88x_lin_2396484bp_60
WT_P-X33	contig_4_103x_lin_137993bp_58.4
WT_P-X33	contig_1_87x_lin_2916224bp_54.7
WT_P-X33	contig_10_85x_lin_1753055bp_58.8

5.5 AWS S3 Bucket Access Guide

5.5.1 Linux/Ubuntu OS using the AWS CLI

1. Install AWS CLI: <https://aws.amazon.com/cli/>.
2. Configure AWS CLI using the following command:

```
aws configure
```

Enter the following when prompted:

- **Access Key ID:** AKIAWOAVSBU2YLWRE4ZT
- **Secret Access Key:** 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

3. To list the bucket contents, run the following command:

```
aws s3 ls s3://cmd00707 --region eu-north-1
```

4. To download a file from the bucket, run the following command:

```
aws s3 cp s3://cmd00707/your-file-name ./
```

5. To recursively download all files in the S3 bucket to your local directory, run the following command:

```
aws s3 cp s3://cmd00707/ ./ --recursive
```

5.5.2 Windows OS using WinSCP

1. Download and install WinSCP from <https://winscp.net>.
2. Open WinSCP and click on 'New Session'.
3. Select 'Amazon S3' as the protocol.
4. Enter the following details:
  - **Host name:** s3.amazonaws.com
  - **Access Key ID:** AKIAWOAVSBU2YLWRE4ZT
  - **Secret Access Key:** ztu5nRxA1oRWyo3GawfPd15y9ae67BLStoqvBwVR
5. Connect and navigate to your bucket: cmd00707 to browse or download data.

5.5.3 Mac OS using Cyberduck

1. Download and install Cyberduck from <https://cyberduck.io>.
2. Open Cyberduck and click on 'Open Connection'.
3. Select 'Amazon S3' from the protocol dropdown.
4. Enter the following details:
  - **Server:** s3.amazonaws.com
  - **Access Key ID:** AKIAWOAVSBU2YLWRE4ZT
  - **Secret Access Key:** ztu5nRxA1oRWyo3GawfPd15y9ae67BLStoqvBwVR
5. Click 'Connect' to access the S3 bucket.
6. Navigate to your bucket: cmd00707 to browse or download data.

References

- 1 Sereika M, Kirkegaard RH, Karst SM, Michaelsen TY, Sørensen EA, Wollenberg RD & Albertsen M (2022) [Oxford nanopore R10.4 long-read sequencing enables the generation of near-finished bacterial genomes from pure cultures and metagenomes without short-read or reference polishing](#). *Nature Methods* **19**, 823–826.
- 2 Koren S & Phillippy AM (2015) [One chromosome, one contig: Complete microbial genomes from long-read sequencing and assembly](#). *Current Opinion in Microbiology* **23**, 110–120.
- 3 Manni M, Berkeley MR, Seppey M & Zdobnov EM (2021) [BUSCO: Assessing genomic data quality and beyond](#). *Current Protocols* **1**, e323.
- 4 Manni M, Berkeley MR, Seppey M, Simão FA & Zdobnov EM (2021) [BUSCO update: Novel and streamlined workflows along with broader and deeper phylogenetic coverage for scoring of eukaryotic, prokaryotic, and viral genomes](#). *Molecular Biology and Evolution* **38**, 4647–4654.
- 5 Gostinčar C (2020) [Towards genomic criteria for delineating fungal species](#). *Journal of Fungi* **6**, 1–18.
- 6 Steinig E & Coin L (2022) [Nanoq: Ultra-fast quality control for nanopore reads](#). *Journal of Open Source Software* **7**, 2991.
- 7 Kolmogorov M, Yuan J, Lin Y & Pevzner PA (2019) [Assembly of long, error-prone reads using repeat graphs](#). *Nature Biotechnology* **37**, 540–546.
- 8 Kolmogorov M, Bickhart DM, Behsaz B, Gurevich A, Rayko M, Shin SB, Kuhn K, Yuan J, Polevikov E, Smith TPL & Pevzner PA (2020) [metaFlye: scalable long-read metagenome assembly using repeat graphs](#). *Nature Methods* **17**, 1103–1110.
- 9 Lin Y, Yuan J, Kolmogorov M, Shen MW, Chaisson M & Pevzner PA (2016) [Assembly of long error-prone reads using de Bruijn graphs](#). *Proceedings of the National Academy of Sciences of the United States of America* **113**, E8396–E8405.
- 10 Wick RR, Schultz MB, Zobel J & Holt KE (2015) [Bandage: Interactive visualization of de novo genome assemblies](#). *Bioinformatics* **31**, 3350–3352.
- 11 Mikheenko A, Prjibelski A, Saveliev V, Antipov D & Gurevich A (2018) [Versatile genome assembly evaluation with QUAST-LG](#). *Bioinformatics* **34**, i142–i150.
- 12 Gabriel L, Brůna T, Hoff KJ, Ebel M, Lomsadze A, Borodovsky M & Stanke M (2024) [BRAKER3: Fully automated genome annotation using RNA-seq and protein evidence with GeneMark-ETP, AUGUSTUS and TSEBRA](#). *Genome Research* **34**, 769–777.
- 13 Pertea G (2020) [GFF utilities: GffRead and GffCompare](#). *F1000Research* **9**, 304.
- 14 Cantalapiedra CP, Hernández-Plaza A, Letunic I, Alfaro M, Blasco R & Huerta-Cepas J (2021) [eggNOG-mapper v2: Functional annotation, orthology assignments, and domain prediction at the metagenomic scale](#). *Molecular Biology and Evolution* **38**, 5825–5829.
- 15 Huerta-Cepas J, Forslund K, Coelho LP, Szklarczyk D, Jensen LJ, Mering C von & Bork P (2019) [eggNOG 5.0: A hierarchical, functionally and phylogenetically annotated orthology resource based on 5090 organisms and 2502 viruses](#). *Nucleic Acids Research* **47**, D309–D314.
- 16 Buchfink B, Reuter K & Drost HG (2021) [Sensitive protein alignments at tree-of-life scale using DIAMOND](#). *Nature Methods* **18**, 366–368.
- 17 Liber JA, Bonito G & Benucci GMN (2021) [CONSTAX2: Improved taxonomic classification of environmental DNA markers](#). *Bioinformatics* **37**, 3941–3943.
- 18 Nilsson RH, Larsson KH, Taylor AFS, Bengtsson-Palme J, Jeppesen TS, Schigel D, Kennedy P, Picard K, Glöckner FO, Tedersoo L, Saar I, Kõljalg U & Abarenkov K (2019) [The UNITE database for molecular identification of fungi: Handling dark taxa and parallel taxonomic classifications](#). *Nucleic Acids Research* **47**, D259–D264.
- 19 Pruesse E, Peplies J & Glöckner FO (2012) [SINA: Accurate high-throughput multiple sequence alignment of ribosomal RNA genes](#). *Bioinformatics* **28**, 1823–1829.
- 20 Quast C, Pruesse E, Yilmaz P, Gerken J, Schweer T, Yarza P, Peplies J & Glöckner FO (2013) [The SILVA ribosomal RNA gene database project: Improved data processing and web-based tools](#). *Nucleic Acids Research* **41**, 590–596.
